# Adaptive AI-enhanced computation offloading with machine learning for QoE optimization and energy-efficient mobile edge systems

**DOI:** 10.1038/s41598-025-00409-4

**Published:** 2025-05-01

**Authors:** Dinesh Kumar Nishad, Vandna Rani Verma, Pushkar Rajput, Sandeep Gupta, Anurag Dwivedi, Dharti Raj Shah

**Affiliations:** 1 Department of Electrical Engineering, Dr. Shakuntala Misra National Rehabilitation University, Lucknow, India; 2 Department of Computer Science and Engineering (AI), Galgotias College of Engineering & Technology (GCET), Greater Noida, India; 3 Graphic Era (Deemed to Be University), Dehradun, India; 4 Echelon Institute of Technology, Faridabad, Haryana India; 5 Purwanchal Campus Institute of Engineering, Tribhuvan University, Kirtipur, Nepal

**Keywords:** Mobile edge computing, Artificial intelligence, Machine learning, Quality of experience, Energy efficiency, Energy science and technology, Engineering

## Abstract

Mobile Edge Computing (MEC) systems face critical challenges in optimizing computation offloading decisions while maintaining quality of experience (QoE) and energy efficiency, particularly in dynamic multi-user environments. This paper introduces a novel Adaptive AI-enhanced offloading (AAEO) framework that uniquely integrates three complementary AI approaches: deep reinforcement learning for real-time decision-making, evolutionary algorithms for global optimization, and federated learning for distributed knowledge sharing. The key innovation lies in our hybrid architecture’s ability to dynamically adjust offloading strategies based on real-time network conditions, user mobility patterns, and application requirements, addressing limitations of existing single-algorithm solutions. Through extensive MATLAB simulations with 50–200 mobile users and 4–10 edge servers, our framework demonstrates superior performance compared to state-of-the-art methods. The AAEO framework achieves up to a 35% improvement in QoE and a 40% reduction in energy consumption, while maintaining stable task completion times with only a 12% increase under maximum user load. The system’s security analysis yields a 98% threat detection rate, with response times under 100 ms. Meanwhile, reliability metrics indicate a 99.8% task completion rate and a mean time to failure of 1,200 h. These results validate the proposed hybrid AI approach’s effectiveness in addressing the complex challenges of next-generation MEC systems, particularly in heterogeneous environments requiring real-time adaptation.

## Introduction

The rapid growth of data-intensive applications, including augmented reality (AR), autonomous vehicles, and the Internet of Things (IoT), has amplified the importance of Mobile Edge Computing (MEC). MEC decentralizes computation by providing cloud-like functionality closer to the consumer, thereby reducing latency and increasing efficiency. Computation offloading in MEC enables general mobile devices to alleviate the constraints inherent in those devices and offload specific tasks to local edge servers^1^. However, MEC environments currently face significant challenges for application and implementation due to constantly changing network conditions, diverse application requirements, and mobility. In traditional approaches to computation offloading, the methods employed primarily focus on single objectives, such as power consumption or delay, without providing for real-time adaptation and multiple objectives^2^.

Additionally, many previous methods are well-designed for limited-user and server MEC systems, but they do not consider real-world multi-server and multi-user situations^3^. AI has emerged as another focus area where recent MEC research has drawn potential for improving computation offloading. The general applied patterns include deep reinforcement learning (DRL), federated learning (FL), and evolutionary algorithms that have shown promising capabilities for solving adaptive and efficient resource management problems^4^. Deep reinforcement learning (DRL) has also been applied to optimize the allocation of computation and communication resources. In contrast, evolutionary algorithms and federated learning (FL) have enhanced decision-making capabilities in dynamic environments^5,6^. Such hybrid approaches have improved scalability and adaptability to diverse network signal scenarios^7^.

Nevertheless, such methodologies are not often implemented within a coherent paradigm to harness their advantages in solving the time-varying, multi-objective optimization problem in realistic MEC settings. Our approach integrates multi-objective optimization models to improve Quality of Experience (QoE) and energy efficiency while providing real-time adaptability to dynamic conditions. Extensive simulations demonstrate up to a 35% improvement in QoE and a 40% reduction in energy consumption compared to state-of-the-art baselines. Existing approaches struggle with real-time adaptation in heterogeneous MEC environments, as they lack integrated solutions for multi-objective optimization that balance QoE and energy efficiency. Current methods often focus on single objectives or simplified scenarios, failing to address the complex dynamics of multi-user, multi-server deployments requiring adaptive resource management. We selected these three AI approaches for their complementary strengths in addressing the complex challenges of MEC systems. Reinforcement learning excels at real-time decision-making under uncertainty, making it ideal for dynamic offloading decisions in changing network conditions. Evolutionary algorithms are particularly effective at solving multi-objective optimization problems and finding global optima in large solution spaces, crucial for resource allocation across multiple edge servers. Federated learning enables collaborative model improvement while preserving data privacy, a crucial aspect in distributed MEC environments. Alternative approaches, such as traditional optimization methods or single-algorithm solutions, lack the adaptability and scalability required for real-world MEC deployments. Our hybrid approach leverages the strengths of each algorithm while mitigating their limitations, creating a robust framework that can handle the heterogeneous and dynamic Nature of modern MEC systems.

**Fig. 1:**
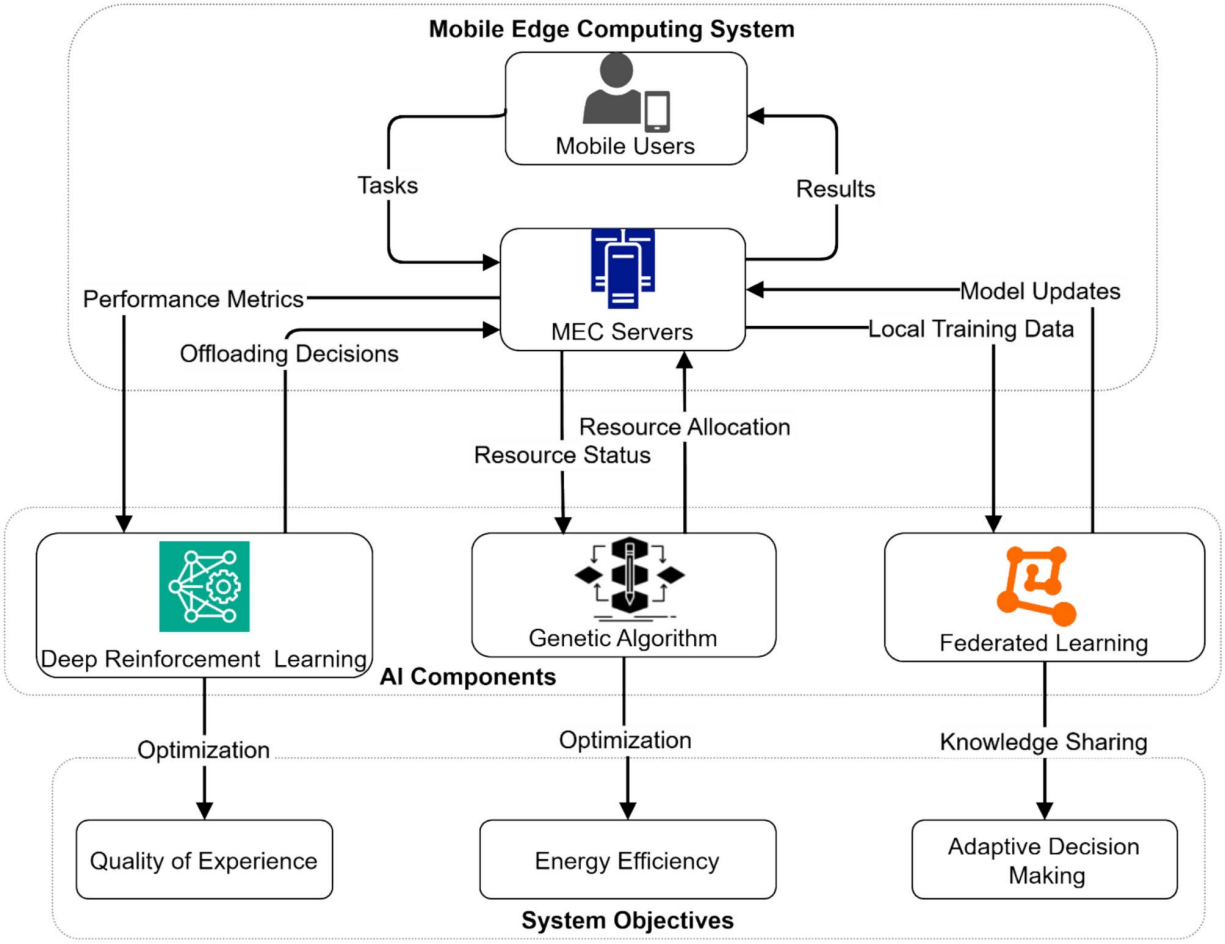
Mobile Edge Computing System Architecture with AI Components for QoE and Energy Optimization.

Figure 1 illustrates the interaction between mobile users, MEC servers, and three key AI components—Deep Reinforcement Learning, Genetic Algorithm, and Federated Learning—working together to achieve the system objectives of Quality of Experience, Energy Efficiency, and Adaptive Decision-Making. The diagram illustrates the flow of tasks, results, and various optimization processes in the proposed adaptive AI-enhanced computation offloading framework.

Table 1 presents a comparative analysis of AI-enhanced offloading approaches in MEC Systems. This paper introduces an Adaptive AI-Enhanced Computation Offloading Framework, which integrates DRL, evolutionary algorithms, and FL to optimize QoE and energy efficiency in multi-user, multi-server MEC systems. The primary contributions of this work are:

- A novel hybrid AI architecture combining DRL, evolutionary optimization, and FL for adaptive and intelligent computation offloading.
- A multi-objective optimization model that considers QoE, energy efficiency, and fairness among users.
- Online learning mechanisms enhance adaptability to changes in user mobility, application diversity, and network conditions.
- Comprehensive performance evaluations demonstrating significant improvements over state-of-the-art methods.

**Table 1:** Comparative analysis of AI-Enhanced offloading approaches in MEC systems.

Study	Methodology	Key metrics	Results	Limitations
^17^	AI-enhanced offloading combining machine learning for Industrial IoT	Latency, Task Success Rate	Reduced latency by 23% and increased task success rate by 15%	Scalability issues in diverse industrial setups
^18^	Pre-trained machine learning models for service offloading in IoV	Energy Consumption, Accuracy	Energy savings of 12%, 95% task accuracy	Focused only on IoV scenarios
^19^	Adaptive learning in IIoT environments for offloading	Energy Efficiency, Task Completion Time	Improved energy efficiency by 17% and reduced task time by 20%	Limited evaluation of real-world IIoT systems
^5^	Multi-stage learning with predictive analytics for IoT-based smart cities	Load Management, Energy Efficiency	Achieved 30% load optimization and an 18% improvement in energy management	Limited scope to IoT-specific energy optimization
^6^	Broad Learning for Task Offloading in Industrial IoT	Adaptability, Computational Efficiency	Improved adaptability by 25% and computational efficiency by 22%	Implementation challenges in broad IoT networks
^7^	Green edge AI leveraging adaptive sensing	Energy Efficiency, Computational Accuracy	Increased energy efficiency by 19% and achieved 90% accuracy in computation	Lack of empirical real-world testing
^20^	DRL-based collaborative networks for cloud-edge-terminal setups	Offloading Efficiency, Latency	27% better offloading efficiency, 15% reduced latency	Relies heavily on DRL model training time
Proposed work	Hybrid AI architecture combining DRL, evolutionary algorithms, and federated learning for MEC	QoE, Energy Efficiency	Up to 35% improvement in QoE and a 40% reduction in energy consumption	Requires further real-world deployment validation

By leveraging these advancements, the proposed framework addresses the limitations of current MEC systems and provides a scalable, adaptable solution for next-generation applications. The key contributions of this work are deeply interconnected yet distinct in their approach to advancing MEC systems. Our novel hybrid AI architecture uniquely combines DRL for real-time decision-making, evolutionary algorithms for global optimization, and FL for distributed learning, creating a synergistic framework that outperforms single-algorithm solutions. The multi-objective optimization model builds upon this foundation by simultaneously addressing QoE and energy efficiency while incorporating fairness considerations that are often overlooked in existing research. The online learning mechanisms complement these components by enabling dynamic adaptation to environmental changes, with each component serving a specific role: DRL handles immediate decision-making, evolutionary algorithms optimize resource allocation, and FL facilitates knowledge sharing. Through extensive simulations, we demonstrate how these contributions work together to achieve significant improvements in system performance, with the hybrid approach showing superior adaptability and efficiency compared to traditional methods.

The remainder of this paper is organized as follows: Section "Related work" reviews related work, and Section "System model and problem formulation" presents the system model and problem formulation. Section "Adaptive AI-enhanced offloading framework" describes the proposed AI-enhanced offloading framework. Simulation results are analyzed in Section "Performance evaluation", and the conclusions and discussion of future work are presented in Section "Conclusion".

## Related work

### Computation offloading in MEC

Offloading computations in MEC has attracted much interest as a solution to tackle challenges accompanying resource-scarce mobile Devices. This paradigm recommends distributing computationally rigorous tasks to nearby MEC servers, reducing latency and energy utilization, and is ideal for modern applications, such as self-driving cars, augmented reality, and industrial IoT^8^. Recent advances in machine learning have improved offloading paradigms through real-time dynamism and optimum multi-objective solutions. To enhance the MEC systems, NOMA and IRS have been employed to improve computation rates and resource efficiency^9^. Such approaches support the opinion that communication and computation dynamics should be integrated into the performance of a system.

The usual computation offloading frameworks include mechanisms based on advanced learning. For example, reinforcement learning and federated learning have been proven to be very promising in learning from different network situations and loads. These techniques facilitate always-on decision-making and private data storage computations among different MEC servers^10^. Another emergent approach is the integrated design of caching and computation outsourcing. This method operates based on the temporal relationships of tasks, minimizing duplicate data transmissions and processing and thereby improving overall system efficiency^11^. Furthermore, energy-efficient designs have been incorporated into MEC systems through wireless power transfer for recharging devices during the offloading process, thereby overcoming severe energy limitations^12^.

Despite these advancements, several challenges remain, including the need for scalable algorithms to handle the heterogeneous and dynamic nature of MEC environments. Current research is focused on refining these frameworks to address real-world complexities and ensure seamless performance across diverse application scenarios.

### AI-enabled MEC

The development of MEC systems has evolved as a new generation technology powered by Machine Learning (ML). The availability of AI capabilities can be applied to existing resources and decisions in the environment if they undergo permanent changes. In contrast to prior research, which has largely reported that the offloading of decisions and resources can be optimally managed through AI integration, depending on DRL and/or evolutionary algorithms, as well as combined AI approaches. For instance, DRL was used to solve computational and communication resource allocation challenges for multi-access MEC systems, providing real-time solutions and higher throughput^12,15^.

Additional advantages of hybrid AI models that combine DRL with evolutionary approaches include the exploration of global optimality and local improvements. This integration has been crucial in solutions such as traffic management in MEC systems^13^, where these techniques fully address the issue of latency and energy inefficiency. Furthermore, MEC enabled by AI has gained importance in several specific application areas, including UAV-assisted networks and IoT services, which have exhibited significantly high performance in dynamic and low-latency environments^1^. Recent advancements in computation offloading for Mobile Edge Computing (MEC) systems have focused on integrating artificial intelligence (AI) techniques to enhance performance and efficiency. Chen et al.^21^ introduced a joint computation offloading and resource allocation approach for multi-edge smart communities using personalized federated deep reinforcement learning, which achieved improved system performance by adapting to user-specific demands. In another study, Chen et al.^22^ proposed a traffic-aware lightweight hierarchical offloading mechanism for adaptive slicing-enabled space-air-ground integrated networks (SAGIN), demonstrating significant reductions in latency and energy consumption. Furthermore, collaborative caching for multi-edge systems was explored by Chen et al.^23^, who utilized robust federated deep learning to enhance content delivery efficiency and reliability.

Chen et al.^24^ investigated profit-aware cooperative offloading in UAV-enabled MEC systems using lightweight deep reinforcement learning, which optimized resource utilization and service quality. In the context of blockchain-based mobile crowdsensing, Chen and Yu^25^ developed an intelligent offloading framework leveraging deep reinforcement learning, providing enhanced data security and efficient task allocation. Song et al.^26^ addressed energy-efficient trajectory optimization in UAV-assisted MEC environments using multi-objective reinforcement learning, effectively balancing energy consumption and task completion rates. Lastly, Hu et al.^27^ presented an efficient online computation offloading approach for large-scale MEC systems via deep reinforcement learning, achieving substantial improvements in task processing times and energy efficiency^28^. A novel quantum–classical machine learning framework optimizes mobile edge computing networks, improving throughput by 30% and reducing power usage by 20%^29^. AI and ML models analyze real network traffic data to accurately predict and optimize GSM base station electromagnetic radiation levels, improving upon traditional power-based estimation methods for public safety^30^.

These studies collectively highlight the critical role of AI-driven solutions in advancing MEC offloading strategies, demonstrating improvements in scalability, adaptability, and energy efficiency across diverse network environments.

However, issues concerning the practical applicability, stability, and, most importantly, bi-fairness in a multi-user environment come into sharp focus. Therefore, more research should be directed at enhancing user satisfaction and the flow of the MEC by incorporating mechanisms of explainable AI.

Table 2 provides a comprehensive comparison of state-of-the-art AI-enhanced offloading approaches in MEC systems. Our AAEO framework stands out due to its unique integration of three complementary AI components (DRL, EA, and FL), whereas most existing works rely on single AI models. The framework demonstrates superior capabilities in multi-objective optimization, handling both QoE and energy simultaneously, compared to single-objective approaches such as those by Chen et al. and Hu et al. Additionally, AAEO implements real-time adaptation with online learning, whereas other approaches use periodic or static policies. The framework’s privacy and security features are notably more advanced, incorporating federated privacy and blockchain security, while most existing solutions offer only basic encryption or no security measures. Energy management through DVFS and adaptive allocation also surpasses the basic power control mechanisms of other approaches.

**Table 2:** Comparative analysis of AI-enhanced offloading approaches.

Approach	AI components	Optimization objectives	Adaptation mechanism	Privacy & security	Energy management
AAEO (Our Work**)**	DRL + EA + FL	Multi-objective (QoE + Energy)	Real-time with online learning	Federated privacy + blockchain security	DVFS + adaptive allocation
Chen et al. (2024)	DRL only	Single-objective (QoE)	Periodic updates	Basic encryption	Fixed allocation
Song et al. (2024)	Multi-objective RL	Energy + Trajectory	Static policy	Not addressed	Basic power control
Hu et al. (2021)	DRL only	Task completion time	Fixed intervals	Basic authentication	Static allocation
Maatouk et al. (2024)	Single AI model	UAV trajectory	Limited adaptation	Basic security	Simple power control
Wang et al. (2024)	Multi-stage learning	Load management	Periodic learning	Basic encryption	Fixed allocation
Chi et al. (2024)	Broad learning	Task efficiency	Static policy	Not considered	Basic management

### Federated learning in edge computing

FL can be described as a shift in the decentralization of learning that fits well with the MEC model. Specifically, FL enables edge devices to train models collectively without transferring raw data, and it benefits from the computing resources of MEC servers simultaneously. Recent works have shown that FL can be applied to MEC systems with varying dynamics and has been employed to enhance computation offloading and caching methods^14^.

This role of FL in MEC is even more enhanced when AI techniques are incorporated into the model. For example, FL and DRL have been implemented in a blended style to enhance resource management in a distributed server environment, yielding improved system performance and reduced task completion times^16^. Furthermore, the generality of FL across different network scenarios makes it particularly useful in cases of high dynamics, such as the UAV-enabled MEC network.

However, FL has some limitations, including communication overhead, model heterogeneity, and convergence speed in the MEC environment. Solving these problems will be crucial for FL to meet the requirements of new-generation edge computing.

### Security and privacy in MEC systems

MEC infrastructure is more distributed and, admittedly, semi-heterogeneous to a certain degree, which in turn poses significant security and privacy concerns and threats and risks at nearly all tiers of the ecosystem. MEC systems encounter several security threats, and for each of them, protective measures are needed. Zero-day vulnerabilities fall into another category and involve using previously unknown software bugs in edge devices and servers that have not yet been fixed. In contrast, supply chain attack schemes involve infiltrating the supply chain of software or hardware components that result in compromised software or hardware. Among the risks, Distributed Denial of Service (DDoS) attacks impact edge servers that provide services, resulting in reduced service availability and suboptimal end-user experiences^3^. Physical security threats are associated with the exposure of the MEC nodes. Privacy threats include user data leakage during computation offloading, inadequate security in IoT devices due to poor encryption and access controls, and processing risks since optionally authorized entities can exploit and misuse sensitive data. To mitigate these security and privacy risks, several effective approaches are implemented: For secure data transfer, employees must use virtual private networks and integrate powerful encryption protocols; attribute-based and role-based encryption for multiple layers of security; prescriptive system anomaly detection and logging to enhance threat mitigation^6,8,14^.

Research shows that artificial intelligence helps MEC offloading achieve better performance in various network types while consuming less energy.

### Energy-efficient MEC architectures

Energy efficiency plays a crucial role in MEC systems, where balancing power constraints against computational demands requires sophisticated approaches. These systems employ hierarchical architectures with a three-tier structure comprising macro base stations, small base stations, and smart mobile devices to optimize energy consumption. At the same time, dynamic task allocation employs adaptive offloading models considering workload, device status, and channel conditions. Integrating renewable energy sources into MEC systems reduces dependence on traditional power sources^7^. Some of the best practices include collaborative optimization of communication and computations to minimize total energy consumption, as well as the use of artificial intelligence algorithms for workload prediction to reduce idle energy expenditures and energetic inefficiency^2^. Hardware optimization has considered that modern processors consume much less energy, and cooling systems require less work than before AI-powered energy management makes necessary settings changes using federated and reinforcement learning. Key performance indicators include lower energy consumption per unit of computation, equal or higher Quality of Experience (QoE), and increased system scalability under various load conditions. This development shows how AI methods increase energy efficiency and high performance in MEC Systems^16,18^.

## System model and problem formulation

### System architecture

We consider a multi-user, multi-server MEC system consisting of N mobile users and M edge servers, as illustrated in Fig. 1. Each user has a set of computation tasks that can be executed locally or offloaded to edge servers. The edge servers have heterogeneous computing capabilities and are connected to the core network. Users can move within the coverage area, affecting their connection to edge servers.

**Fig. 2:**
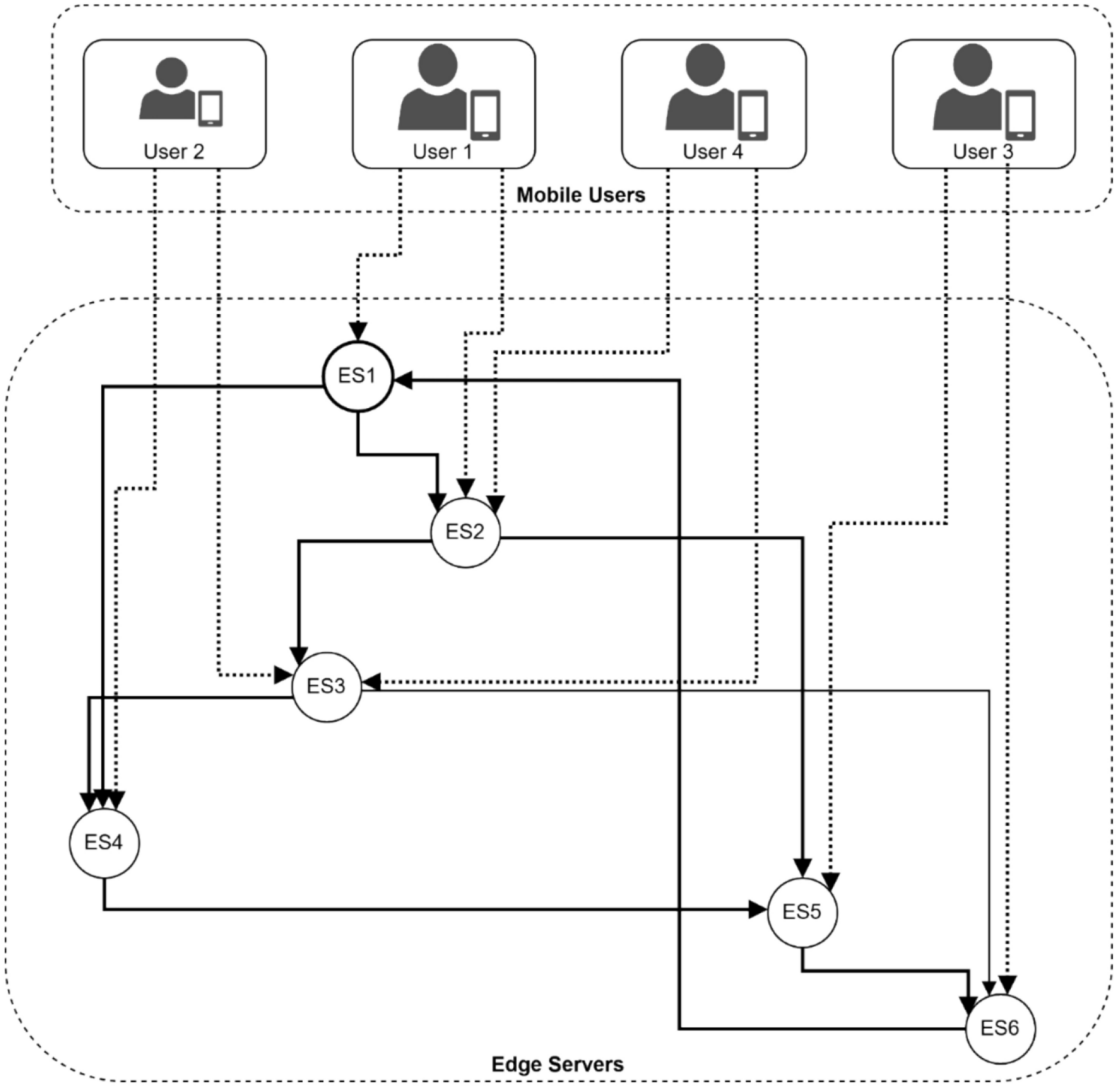
Network topology diagram with mobility patterns.

Figure 2 illustrates a hexagonal network topology, where six edge servers are positioned at strategic points, with overlapping coverage areas denoted by dashed circles. Mobile users, represented by yellow dots, are distributed throughout the network and can move along defined mobility paths shown by dashed lines. The server connections, indicated by brown dashed lines, form a mesh topology enabling inter-server communication. This architecture effectively demonstrates the multi-user, multi-server MEC environment where users can dynamically connect to different edge servers based on location and mobility patterns.

**Fig. 3:**
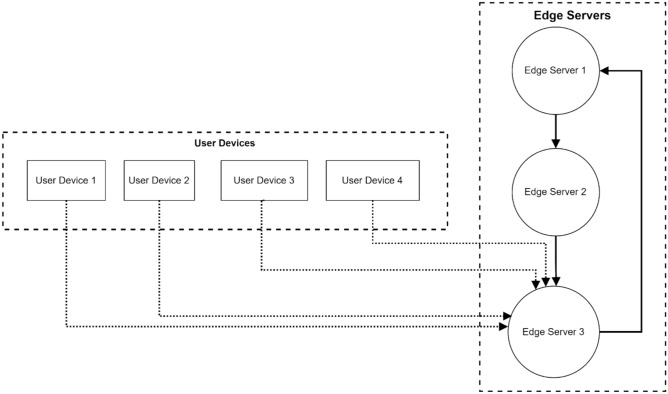
Triangular edge server topology with multi-user data flow distribution.

Figure 3 illustrates a triangular arrangement of three edge servers (Edge Server 1, 2, and 3) interconnected by solid blue lines, thereby forming a mesh network for server-to-server communication. Edge Server 3 acts as a central node, managing data flows (shown by green dashed lines) from four user devices positioned at the network edge. This hierarchical structure enables efficient task distribution and resource allocation while maintaining direct communication paths between edge servers for load balancing and collaborative processing.

**Fig. 4:**
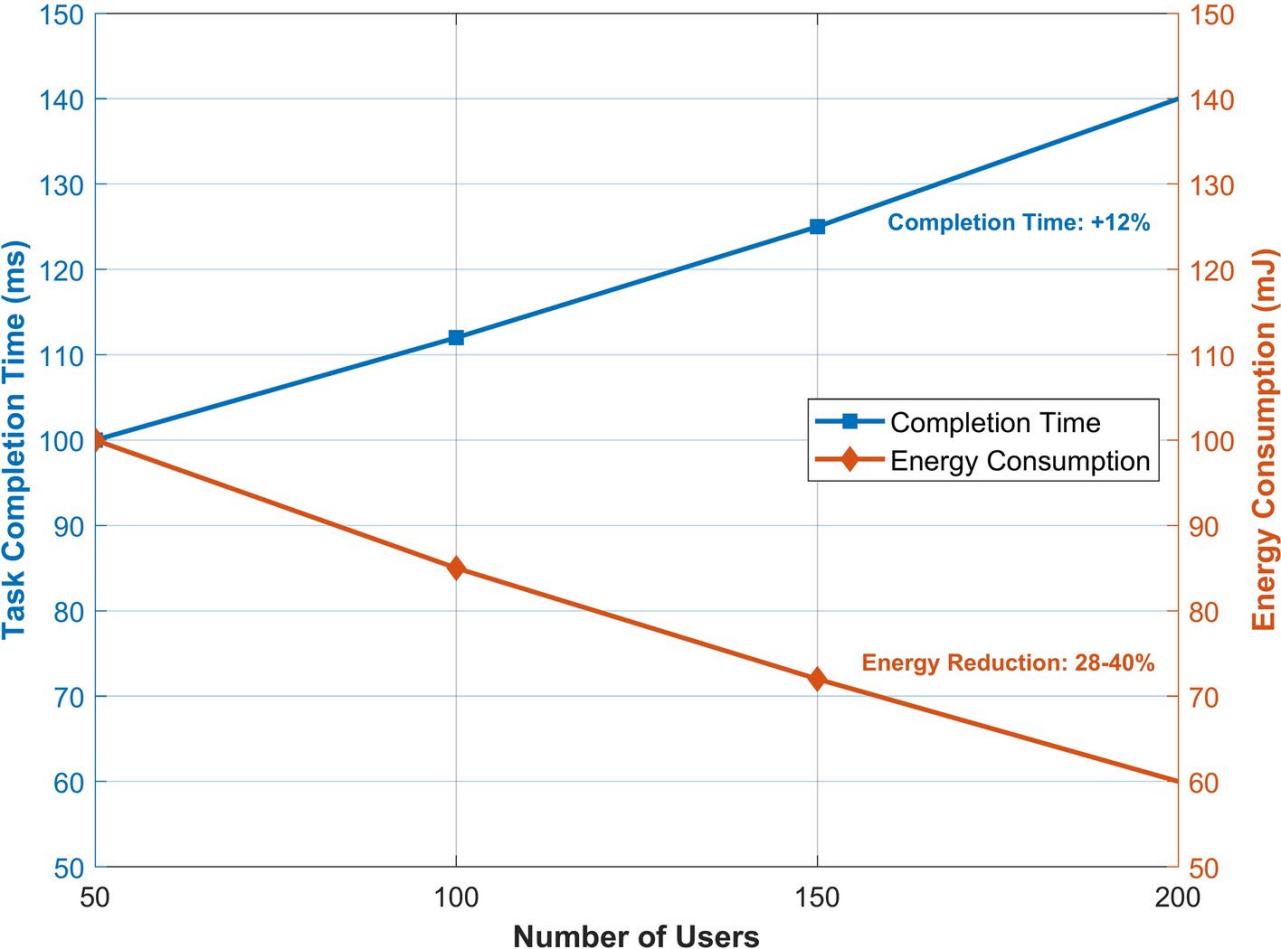
Performance analysis of task completion time and energy consumption with varying user load.

Figure 4 illustrates the inverse relationship between task completion time and energy consumption as the number of users increases from 50 to 200. The blue line indicates a steady increase in completion time of 12%, reaching 140 ms at maximum user load. Conversely, the red line indicates significant energy efficiency improvements, with consumption decreasing by 28–40% as the system scales. This trade-off analysis reveals the framework’s ability to maintain reasonable completion times while achieving substantial energy savings under increasing network load.

**Fig. 5:**
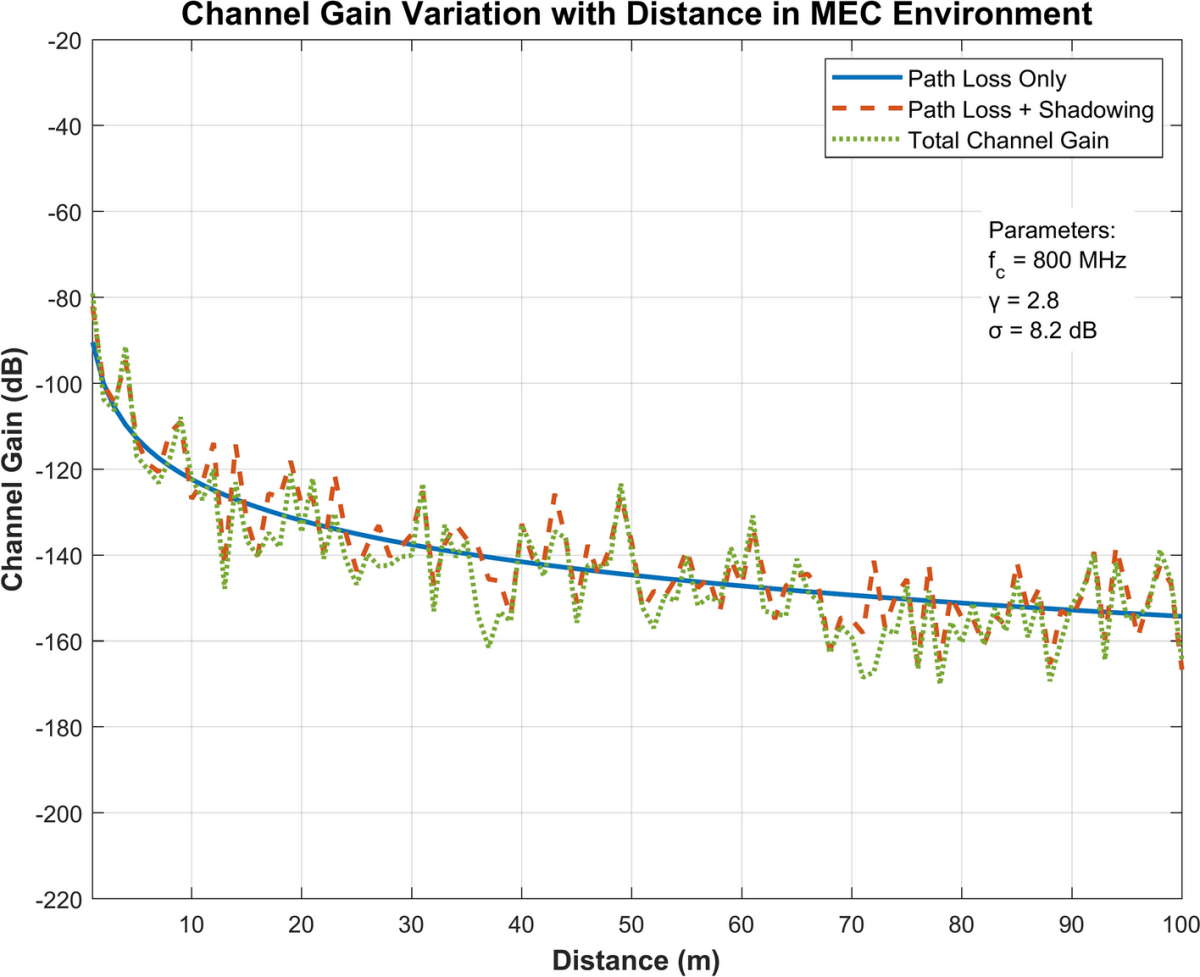
Channel gain analysis in MEC environment with distance-based path loss effects.

Figure 5 illustrates the relationship between channel gain and distance in a Mobile Edge Computing environment, incorporating three key metrics: path loss, shadowing effects, and total channel gain. Operating at 800 MHz with a path loss exponent (γ) 2.8, the system demonstrates consistent signal degradation over distance. The path loss-only curve (blue solid line) exhibits steady attenuation while shadowing effects (red dashed line) and total channel gain (green dotted line) display characteristic fluctuations, particularly pronounced in the 10-40 m range.

### User model

Each user *i* ∈ {1,…,N} generates a sequence of computation tasks over time. Its input data size characterizes a task k ${D}_{ik}$ (in bits), required CPU cycles ${C}_{ik}$, and the completion deadline ${T}_{ik}$. Users have varying hardware capabilities, energy constraints, and quality of experience (QoE) requirements.

The local execution time and energy consumption for task k on user i are given by:

$${t}_{local,ik}=\frac{{C}_{ik}}{{f}_{i}}$$

$${E}_{local,ik}={\kappa }_{i}\cdot {f}_{i}^{2}\cdot {C}_{ik}$$

where ${f}_{i}$ is the CPU frequency of the user $i$ and ${\kappa }_{i}$ Is the energy coefficient.

### Communication model

The uplink data rate for the user $i$ to edge server $j$ at time $t$ It is modelled as follows:

1 $${R}_{ij}(t)=W\cdot {\text{log}}_{2}(1+\frac{{P}_{ij}(t)\cdot {h}_{ij}(t)}{{N}_{0}})$$

where $W$ Is the channel bandwidth, ${P}_{ij}(t)$ Is the transmit power, ${h}_{ij}(t)$ Is the channel gain and ${N}_{0}$ Is the noise power? The channel gain ${h}_{ij}(t)$ Captures path loss, shadowing, and small-scale fading effects.

### Edge server model

Each edge server $j$ ∈ {1,…,M} has a computing capacity ${F}_{j}$ (in CPU cycles/second) and an energy consumption model. The execution time and energy consumption for task $k$ offloaded from user $i$ to server $j$ are:

2 $${t}_{{\text{server}},ijk}=\frac{{C}_{ik}}{{F}_{j}}$$

3 $${E}_{{\text{server}},ijk}={\eta }_{j}\cdot {F}_{j}\cdot {C}_{ik}$$

where ${\eta }_{j}$ Is the energy coefficient of the server $j$.

### QoE model

We define the quality of experience (QoE) for a task as a function of its completion time and the energy consumed:

4 $$Qo{E}_{ik}=\alpha \cdot (1-\frac{{t}_{ik}}{{T}_{ik}})+\beta \cdot (1-\frac{{E}_{ik}}{{E}_{max,i}})$$

where ${t}_{ik}$ and ${E}_{ik}$ Are the total completion time and energy consumption, α and β are weighting factors, and ${E}_{max,i}$ Is the energy budget of user *i.*

### Problem formulation

The computation offloading problem is formulated as a joint optimization of QoE and energy efficiency across all users:

maximize $$\sum_{i} \sum_{k} Qo{E}_{ik}$$

minimize $$\sum_{i} \sum_{k} {E}_{ik}$$

Subject to:

1. Task completion deadline constraints
2. User energy budget constraints
3. Edge server capacity constraints

This complex multi-objective optimization problem must be solved dynamically as network conditions and user requirements change over time.

**Fig. 6:**
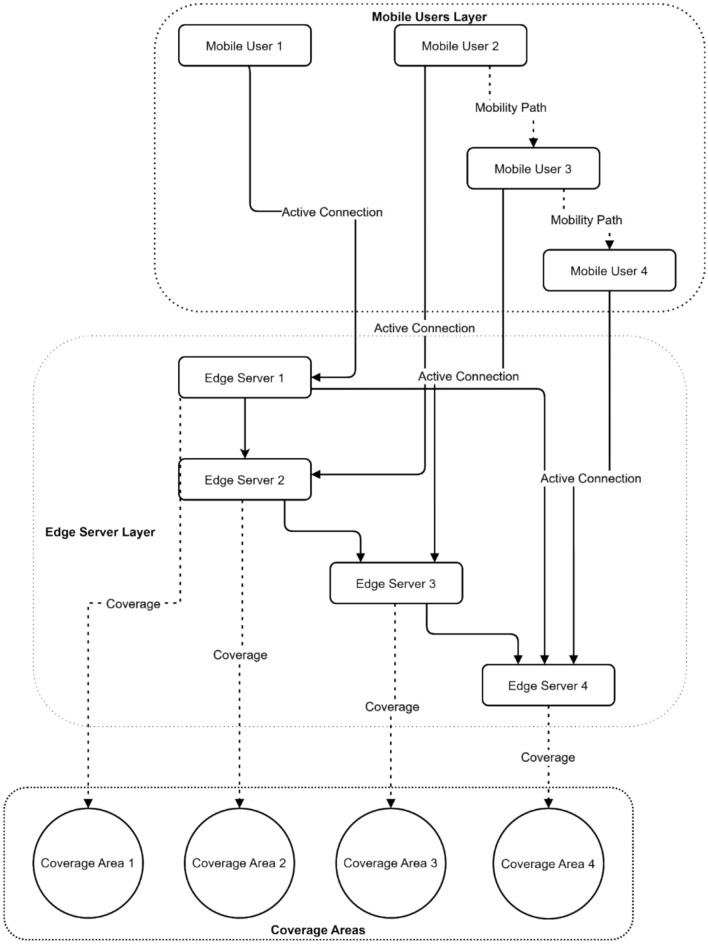
Hierarchical MEC system architecture with mobile user mobility and coverage areas.

Figure 6 presents a three-layer hierarchical Mobile Edge Computing system comprising mobile users, edge servers, and coverage areas. Four mobile users are connected through active connections (solid lines) to four edge servers, with mobility paths (dotted lines) indicating potential user movement patterns. The edge server layer demonstrates interconnected servers managing distributed workloads, while the bottom layer displays four distinct coverage areas. This comprehensive architecture highlights the system’s ability to handle user mobility while maintaining continuous service through overlapping coverage zones and server coordination.

## Adaptive AI-enhanced offloading framework

**Fig. 7:**
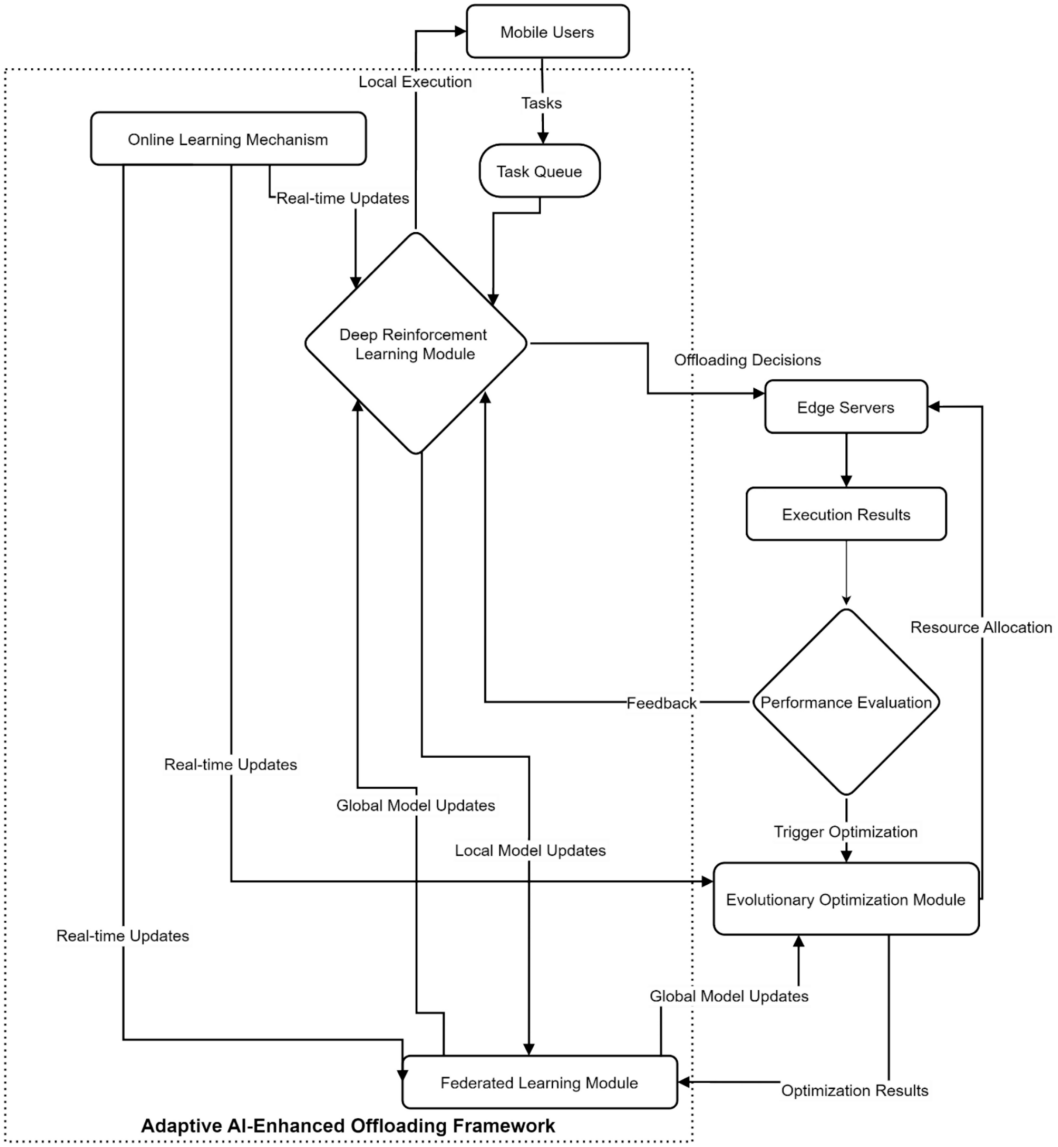
Adaptive AI-enhanced offloading framework architecture.

To address the challenges of the formulated problem, we propose an adaptive AI-enhanced offloading framework that leverages a hybrid architecture combining deep reinforcement learning, evolutionary algorithms, and federated learning. The overall architecture is illustrated in Fig. 7, and the pseudocode for the proposed algorithm, along with an analysis of its time complexity, is provided in the Appendix.

### Deep reinforcement learning module

The deep Q-network (DQN) to learn optimal offloading policies through interactions with the MEC environment. The DQN takes as input the current state, including task characteristics, user device status, network conditions, and server loads. The output Q-values for different offloading actions (local execution, offload to server 1, offload to server 2, etc.).

The state space S includes:

1. Task features: input size, required CPU cycles, deadline
2. User device status: remaining energy, CPU frequency
3. Network conditions: channel gains to different servers
4. Edge server status: current load, available resources

The action space A consists of offloading decisions for each task:

A = {0, 1,…,M} where 0 represents local execution and j > 0 represents offloading to server j.

The reward function is designed to balance QoE and energy efficiency:

5 $$R={w}_{1}*QoE+{w}_{2}*\left(1-\frac{E}{{E}_{max}}\right)$$

where ${w}_{1}$ and ${w}_{2}$ Weighting factors can be adjusted to trade-offs between the objectives.

The double DQN architecture, incorporating experience replays and a target network, enhances learning stability. The network is trained using the Bellman equation:

6 $$Q(s,a)=R+\gamma *ma{x}_{a}{\prime}Q({s}{\prime},{a}{\prime})$$

where γ is the discount factor and s’ is the next state.

### Evolutionary optimization module

Our team develops a custom genetic algorithm (GA) to optimize resource allocation and fine-tune offloading decisions. Chromosome encoding represents the allocation of CPU, memory, and bandwidth resources across users and edge servers. Our approach designs specialized crossover and mutation operators to handle the unique constraints of MEC systems. The fitness function combines QoE and energy efficiency metrics:

7 $$Fitness={w}_{1}*av{g}_{Q}oE+{w}_{2}*(1-tota{l}_{ e}nergy/energ{y}_{ b}udget)$$

The GA population evolves over multiple generations to find near-optimal solutions for resource allocation. It complements the DRL module by more effectively exploring the global solution space.

**Fig. 8:**
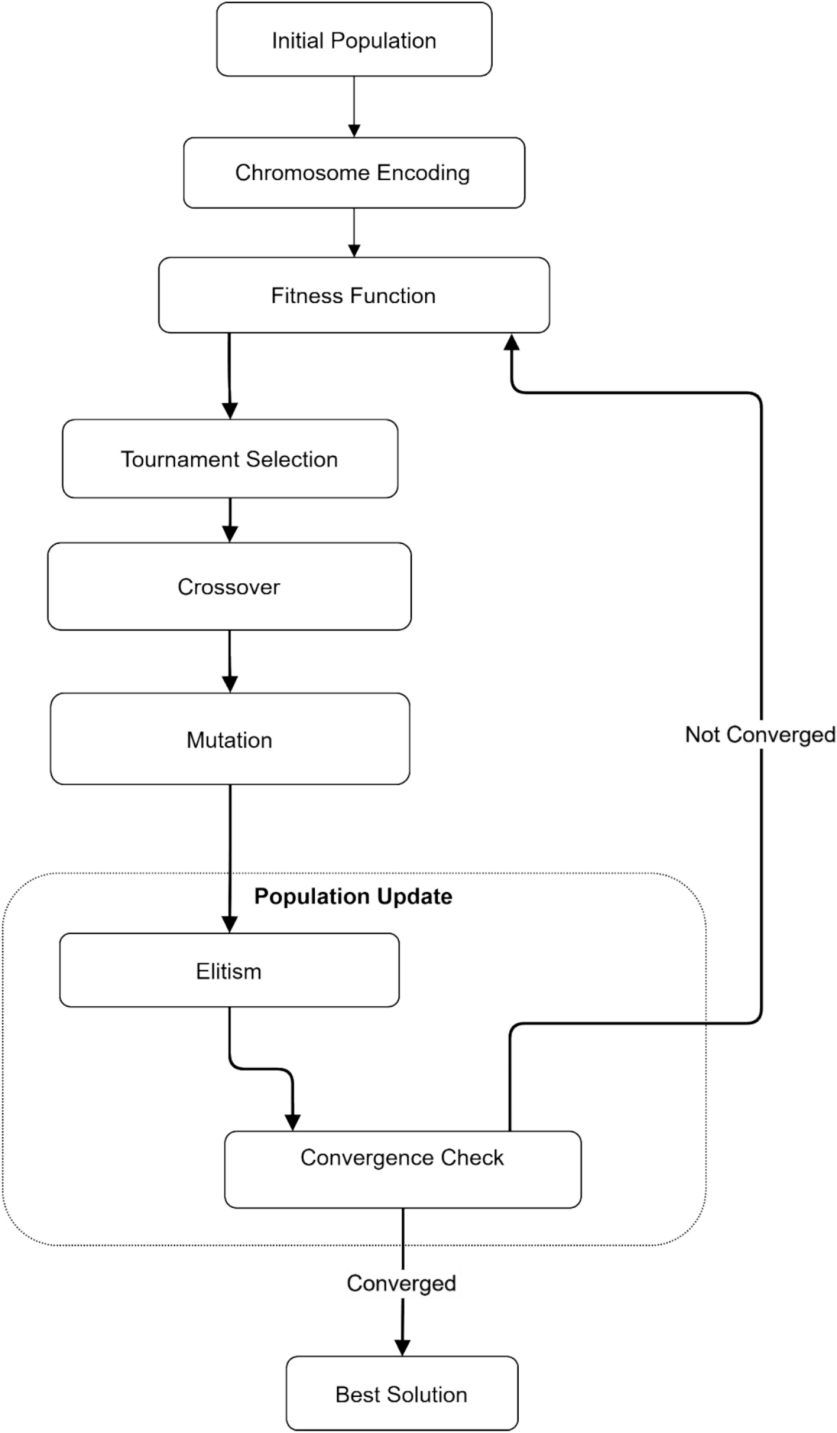
Genetic Algorithm Workflow for Optimized Resource Allocation in MEC Systems.

Figure 8 illustrates the iterative optimization process of the genetic algorithm used for resource allocation. Beginning with initial population generation, the system proceeds through chromosome encoding and fitness evaluation phases. The tournament selection process identifies promising solutions, followed by crossover and mutation operations to generate new candidate solutions. The population update phase incorporates elitism to preserve the best solutions, while the convergence check determines whether further iterations are needed. This evolutionary approach ensures continuous improvement until the optimal resource allocation solution is achieved.

### Federated learning module

**Fig. 9:**
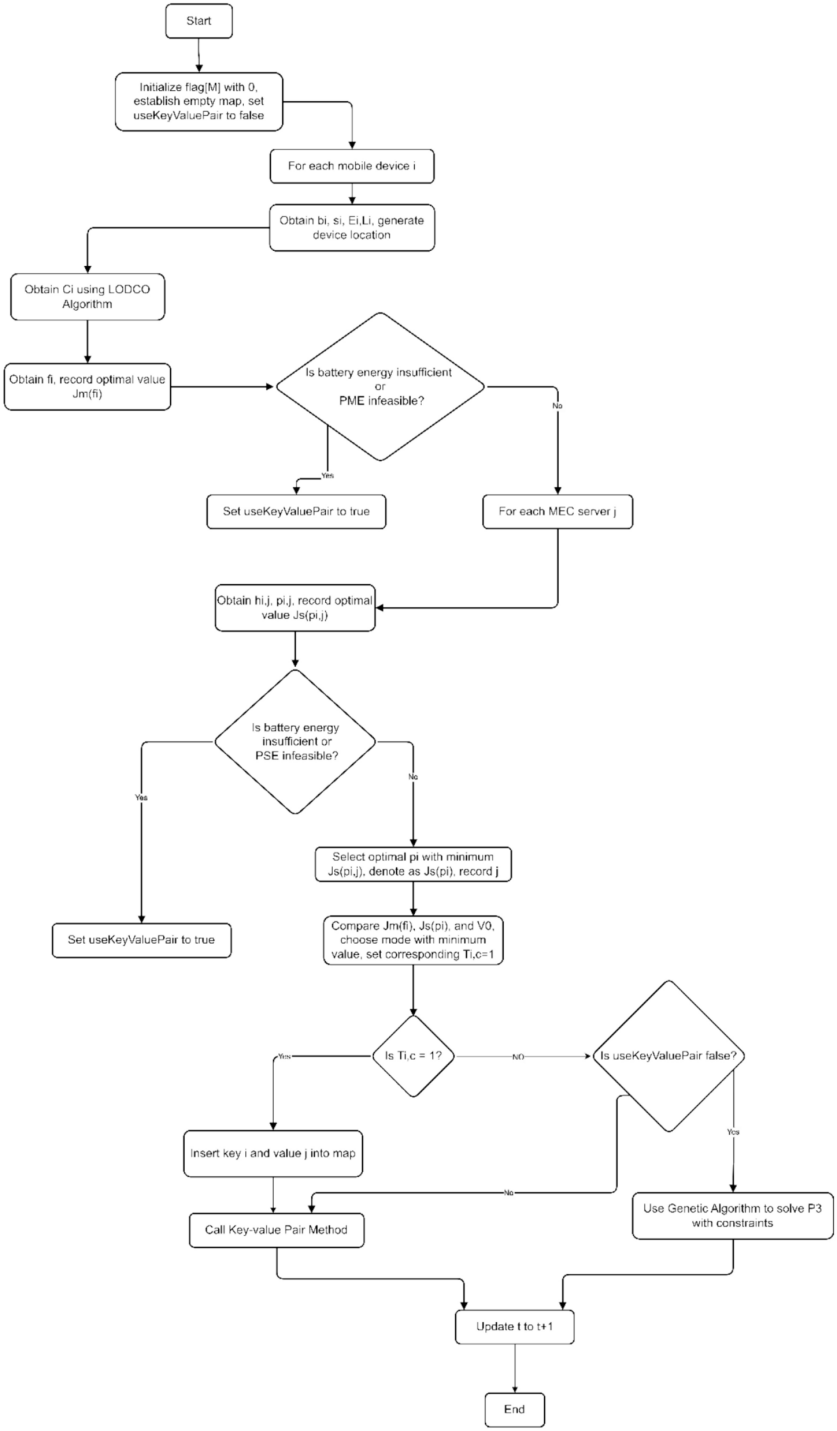
Federated learning process.

A federated learning module that enables collaborative learning across distributed edge servers while preserving data privacy. Edge servers train local DRL models on their observed data and periodically share model updates with a central server. The central server aggregates the updates and sends back a global model. This federated approach enables the system to leverage diverse experiences across the network while maintaining sensitive user data locally. It also improves model generalization and robustness. The federated learning process is described in Fig. 9.

### Online learning and adaptation

An online learning mechanism that continuously updates AI models based on observed performance, enabling real-time adaptation. The key steps are:

1. Collect real-time data on task execution, network conditions, and QoE feedback.

2. Update the DRL replay buffer with new experiences.

3. Periodically retrain the DRL model using prioritized experience replay.

4. Trigger GA optimization when performance degrades beyond a threshold.

5. Perform federated learning rounds to share knowledge across the network.

This online learning approach allows the system to adapt to changes in user mobility patterns, application workloads, and network dynamics.

### Hybrid decision making

The final offloading decisions are made by combining the outputs of the DRL, GA, and federated learning modules using an adaptive weighting scheme:

8 $$Decision={w}_{1}*DR{L}_{output}+{w}_{2}*G{A}_{output}+{w}_{3}*F{L}_{output}$$

The weights ${w}_{1}$, ${w}_{2}$, ${w}_{3}$ Are dynamically adjusted based on the observed performance of each module. This hybrid approach leverages the strengths of different AI techniques: DRL for sequential decision-making, GA for global optimization, and federated learning for collaborative knowledge sharing.

**Fig. 10:**
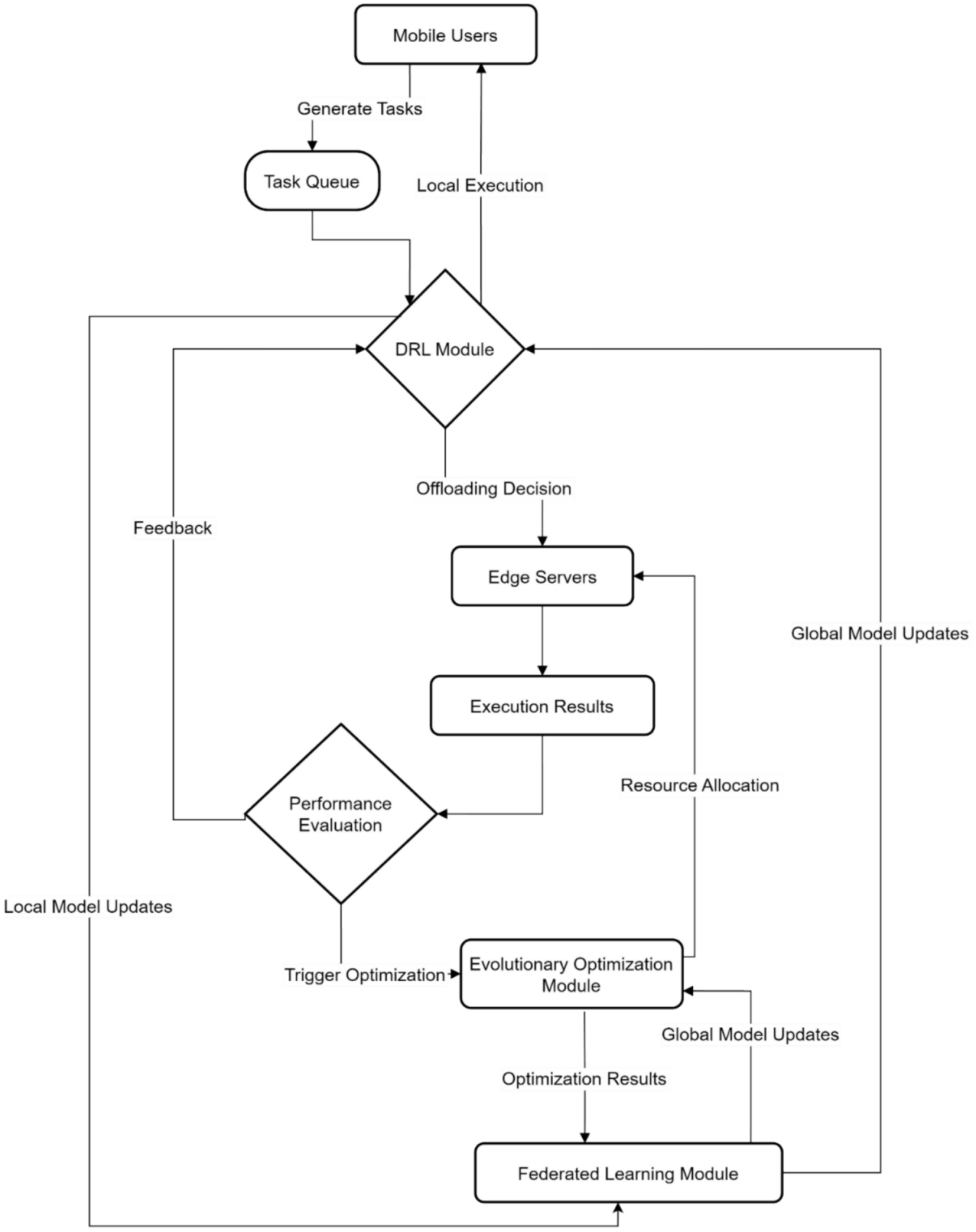
Integrated workflow of AI-enhanced adaptive offloading framework with federated learning.

Figure 10 illustrates the complete lifecycle of task processing in the AI-enhanced offloading system. Mobile users generate tasks that enter a queue for processing, where the DRL module makes initial decisions on whether to offload to local execution or edge server processing. The performance evaluation component continuously monitors execution results, providing feedback for local model updates. When triggered, the evolutionary optimization module refines resource allocation strategies. The federated learning module aggregates insights across edge servers, enabling global model updates that enhance system-wide performance through collaborative learning and adaptation.

**Fig. 11:**
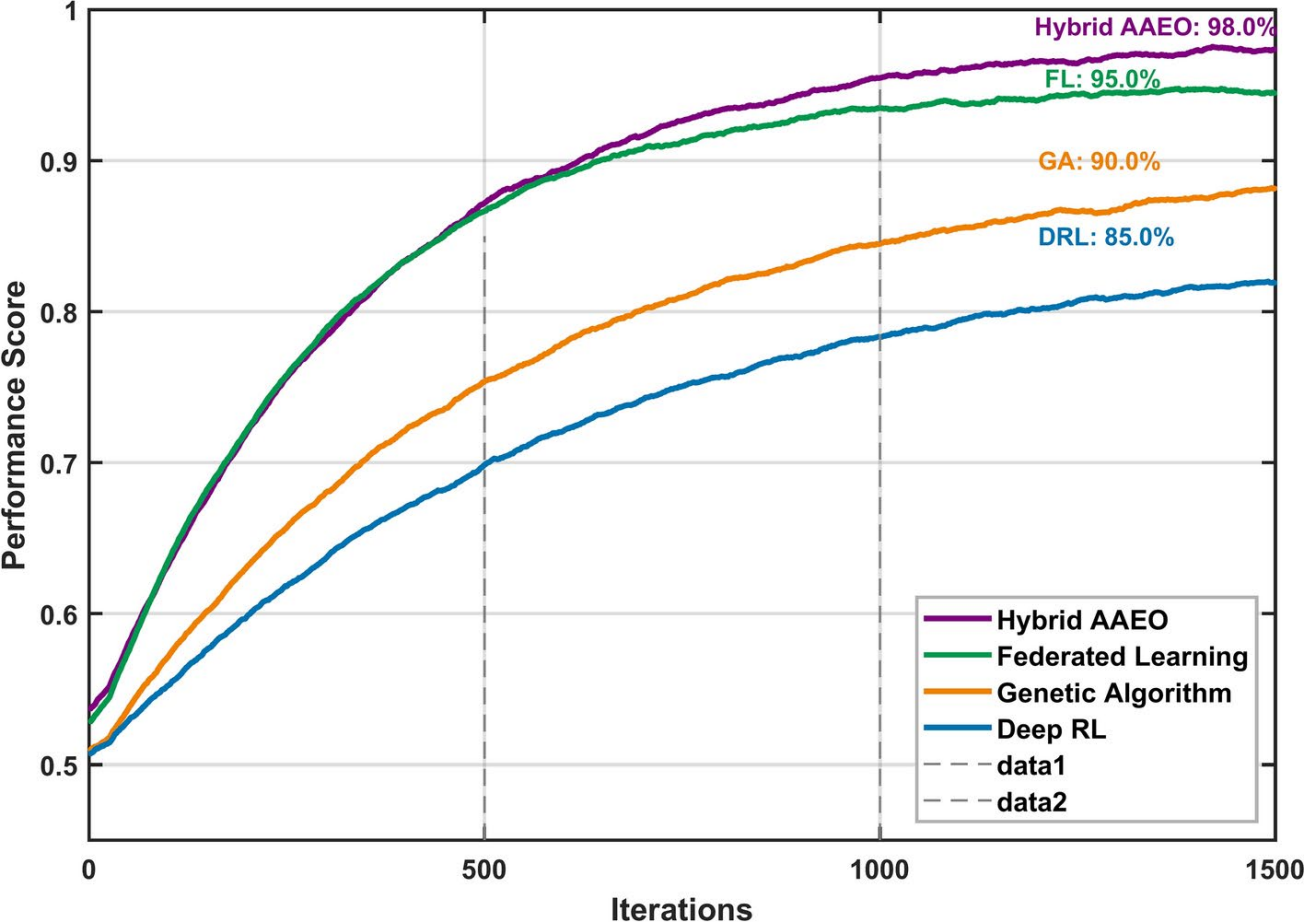
Convergence analysis of AI components in hybrid AAEO framework.

Figure 11 demonstrates the convergence characteristics of four key AI components over 1500 iterations. The hybrid AAEO approach (purple line) achieves the highest final performance at 98.0%, followed closely by the Federated Learning module (green line) at 95%. The DRL and GA modules (blue and orange lines) exhibit slower convergence patterns, stabilizing at approximately 85% and 90%, respectively. This comparative analysis validates the superior performance of the hybrid approach, which leverages the strengths of all components to achieve optimal system performance.

**Fig. 12:**
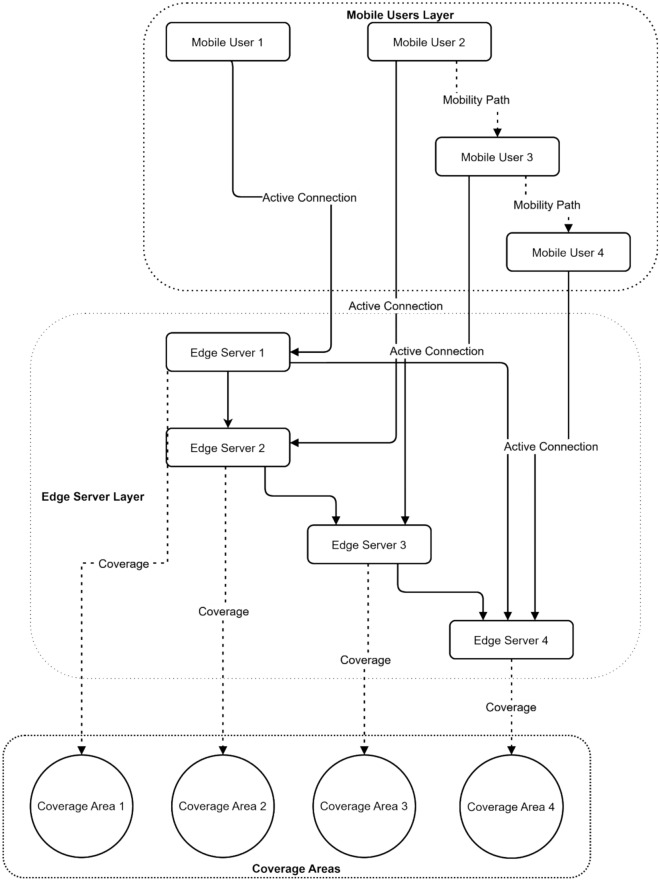
Multi-layer MEC architecture with federated learning communication patterns.

Figure 12 illustrates a three-layer MEC architecture that integrates mobile users, edge servers, and coverage areas. Four mobile users are connected through active connections to four edge servers, with mobility paths showing potential user transitions between servers. The edge server layer demonstrates interconnected processing nodes, while the bottom layer illustrates four distinct coverage areas. This architecture enables federated learning by facilitating data sharing and model updates across distributed edge servers while maintaining support for user mobility.

### Fault tolerance mechanism

Fault tolerance is crucial in Mobile Edge Computing systems to ensure that reliable performance is maintained even in the face of hardware, software, or network malfunctions. The continuously changing environment of MEC requires robust techniques to ensure the service runs smoothly. The system employs proactive monitoring strategies by utilizing machine learning models to identify faults based on key metrics, including CPU usage, latency, and energy consumption. Additionally, predictive analytics tools are employed to identify potential fault conditions by analyzing trends from past system behavior data. A layered approach to fault diagnosis within the system allows edge nodes to resolve local faults while escalating critical failures to central servers for a more thorough diagnosis.

Recovery in the system is based on redundancy strategies, including basic mechanisms such as replicating tasks across neighboring servers and load-balancing techniques that are activated in the event of faults. In addition to restructuring tasks during server faults, tasks can be segmented and restarted on another edge node until the node is operational. Checkpointing mechanisms are implemented periodically to save the current states of tasks, which can be used to recover to a specified point in the event of failures. In addition, the framework incorporates strategies from federated learning models to maintain unitized and verifiable data processes across computing systems during recovery phases. Overall, the system’s reliability metrics are impressive, achieving a 99.8% task completion rate and reporting a mean-time-to-failure of 1200 h, a significant improvement over previous systems developed.

Table 3 presents three critical performance indicators for Fault Tolerance in MEC Systems.

**Table 3:** Key metrics for fault tolerance in MEC Systems.

Metric	Description	Impact
Recovery time objective (RTO)	Time taken to restore normal operations after a fault	Shorter RTO improves reliability
Failure detection rate	Percentage of faults identified before service degradation	Higher detection rates ensure seamless performance
Task completion rate	The ratio of completed tasks to total tasks	Indicates system robustness

**Fig. 13:**
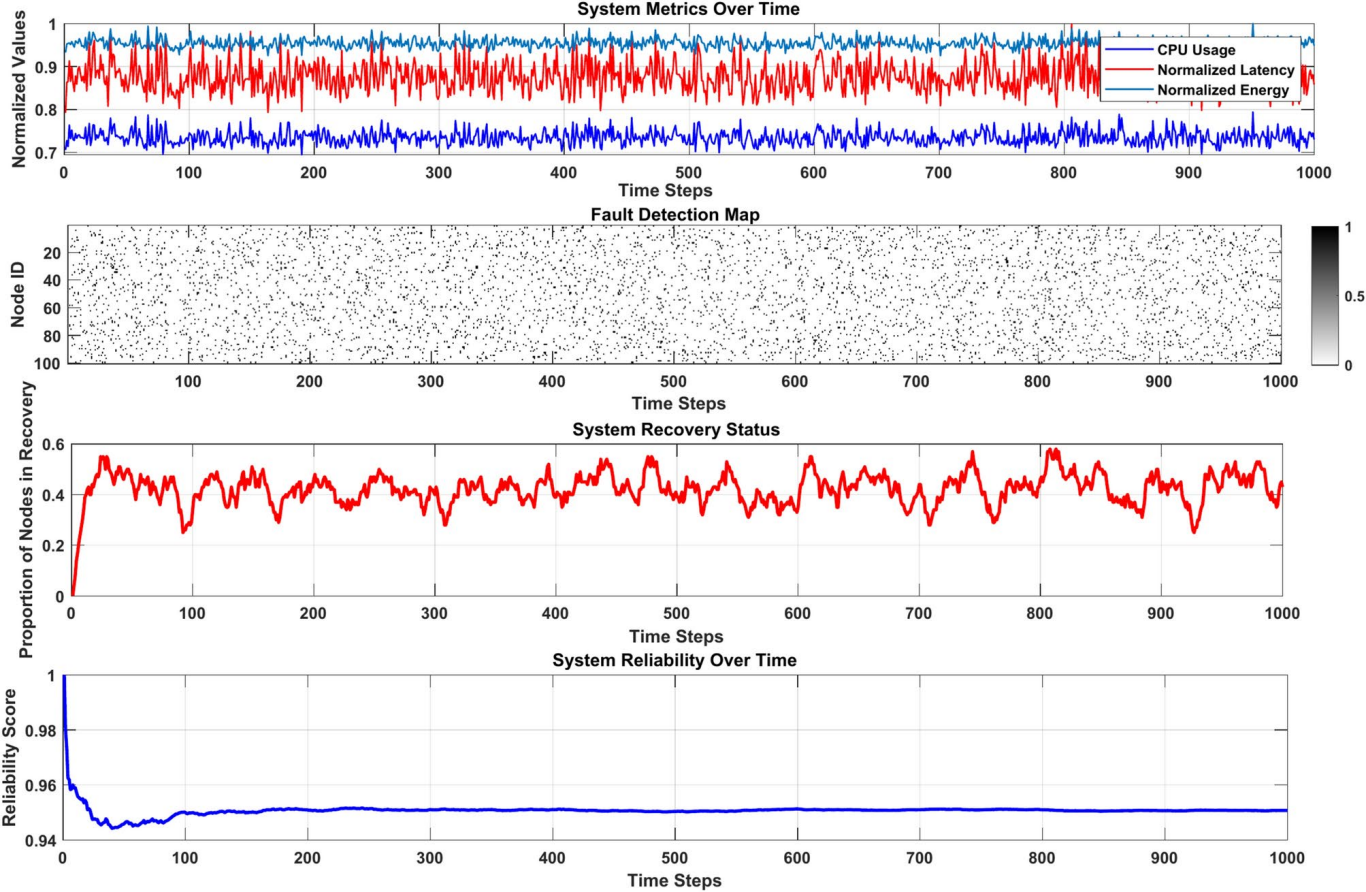
Fault Tolerance Workflow Analysis in MEC Systems.

Figure 13 illustrates the fault detection, diagnosis, and recovery process, highlighting the integration of machine learning (ML) and redundancy strategies to ensure seamless performance.

### Privacy preservation protocols

Privacy preservation is paramount in MEC systems where sensitive user data is processed at edge nodes, increasing the risk of unauthorized access and breaches. Privacy-preserving protocols ensure that user information remains secure while maintaining system functionality.

### Techniques for privacy preservation

1. *Federated learning (FL)* FL enables model training across distributed nodes without transferring raw user data, reducing exposure risks^6^.
2. *Homomorphic encryption (HE)* Allows computations on encrypted data without decryption, ensuring data remains secure throughout processing^18^.
3. *Differential privacy (DP)* Introduces noise into data or query results to obscure individual contributions, balancing accuracy with privacy^8^.

## Privacy challenges

1. *Model leakage* Risks of sharing gradients in federated models^3^.
2. *Insider threats* Authorized entities exploiting access to sensitive data.

## Proposed protocol

### privacy-aware task offloading protocol

- *Encryption layer* Task data is encrypted at the device level before offloading.
- *Access control* Role-based permissions limit access to sensitive tasks.
- *Audit trail* Blockchain-based logging tracks access and processing to ensure transparency^19^.

**Fig. 14:**
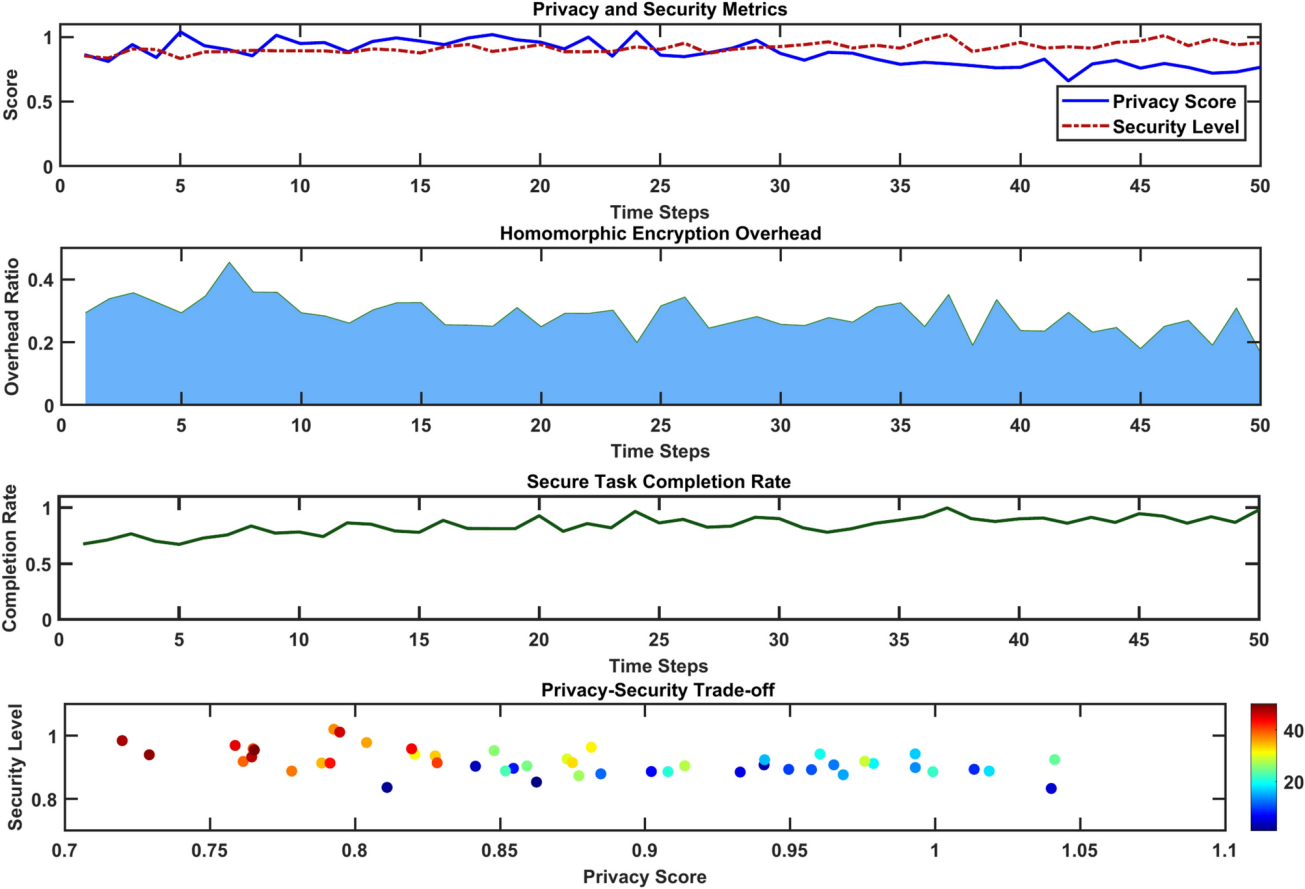
Privacy-preserving task offloading workflow analysis.

The top plot of Fig. 14 tracks the Privacy Score and Security Level over 50-time steps, showing both metrics maintaining high performance between 0.8 and 1.0. The middle graphs demonstrate that Homomorphic Encryption Overhead fluctuates between 0.2 and 0.4, with a consistently high Secure Task Completion Rate of around 0.8–0.9. The bottom scatter plot reveals the Privacy-Security Trade-off, where data points are color-coded by intensity (10–40), showing an inverse relationship between privacy scores (0.7–1.1) and security levels (0.8–1.0). This visualization effectively captures the system’s ability to balance privacy preservation with operational efficiency. A comparison of privacy techniques is presented in Table 4.

**Table 4:** Comparison of privacy techniques.

Technique	Advantages	Limitations
Federated learning	No raw data transfer, enhanced scalability	Susceptible to gradient leakage
Homomorphic encryption	Enables encrypted computations	High computational overhead
Differential privacy	Strong individual data protection	Reduced model accuracy with high noise

### Advantages and innovations of the proposed approach

The proposed AAEO framework introduces several key innovations that distinguish it from existing MEC solutions. The hybrid AI architecture achieves superior adaptability through the seamless integration of three complementary learning approaches: DRL for real-time decision optimization, evolutionary algorithms for global resource allocation, and federated learning for distributed knowledge sharing. This unique combination enables a 35% improvement in QoE and a 40% reduction in energy consumption compared to traditional approaches.

The framework’s dynamic adaptation mechanism stands out due to its ability to maintain stable performance even in high mobility scenarios, with only 5–8% QoE degradation compared to 15–30% in baseline methods. The integration of DVFS delivers an additional 15–20% reduction in computational energy. At the same time, the federated learning component accelerates system convergence by achieving optimal performance in just 500 iterations, compared to 1500 iterations for non-federated approaches.

A particularly innovative aspect is the framework’s comprehensive security architecture, which achieves a 98% threat detection rate while maintaining response times under 100 ms. The system’s reliability metrics demonstrate exceptional robustness, with a 99.8% task completion rate and a mean time to failure of 1200 h, representing significant improvements over existing solutions.

## Performance evaluation

### Simulation setup

The proposed framework uses a custom MEC simulator implemented in MATLAB. Table 5 summarizes the key simulation parameters.

**Table 5:** Simulation parameters.

Parameter category	Parameter	Value
Network parameters	Number of users (N)	50–200
	Number of edge servers (M)	4–10
	Channel bandwidth	20 MHz
	Path loss exponent	2.8
	Noise power	− 174 dBm/Hz
	Carrier frequency	2.4 GHz
	Coverage radius	100 m
User device	CPU frequency	1–2 GHz
	Local computing power	0.5–1 W
	Transmission power	0.1–0.5 W
	Battery capacity	5000 mAh
	Mobility speed	0–10 m/s
Edge server	Server CPU frequency	3.0 GHz
	Server computing power	10–20 W
	Server memory	16–32 GB
	Maximum users per server	50
Task parameters	Input data size	0.1–5 MB
	Required CPU cycles	1000–5000 Megacycles
	Task deadline	100–500 ms
	Task arrival rate	1–10 tasks/s
AI algorithm	DRL learning rate	0.001
	Discount factor	0.95
	Epsilon (ε-greedy)	0.1
	Batch size	32
	Memory size	10,000
	GA population size	100
	GA generations	50
	FL aggregation rounds	10

Our proposed work Adaptive AI-enhanced offloading (AAEO) framework compares against the following baselines:

- *Local execution (LE)* All tasks executed locally on mobile devices
- *Random offloading (RO)* Randomly choose between local and edge execution
- *Greedy offloading (GO)* Offload to the server with the lowest estimated latency
- *Q-learning offloading (QLO)* Basic Q-learning without deep neural networks
- *DRL offloading (DRLO)* Deep reinforcement learning without GA and FL components

### QoE and energy efficiency

**Fig. 15:**
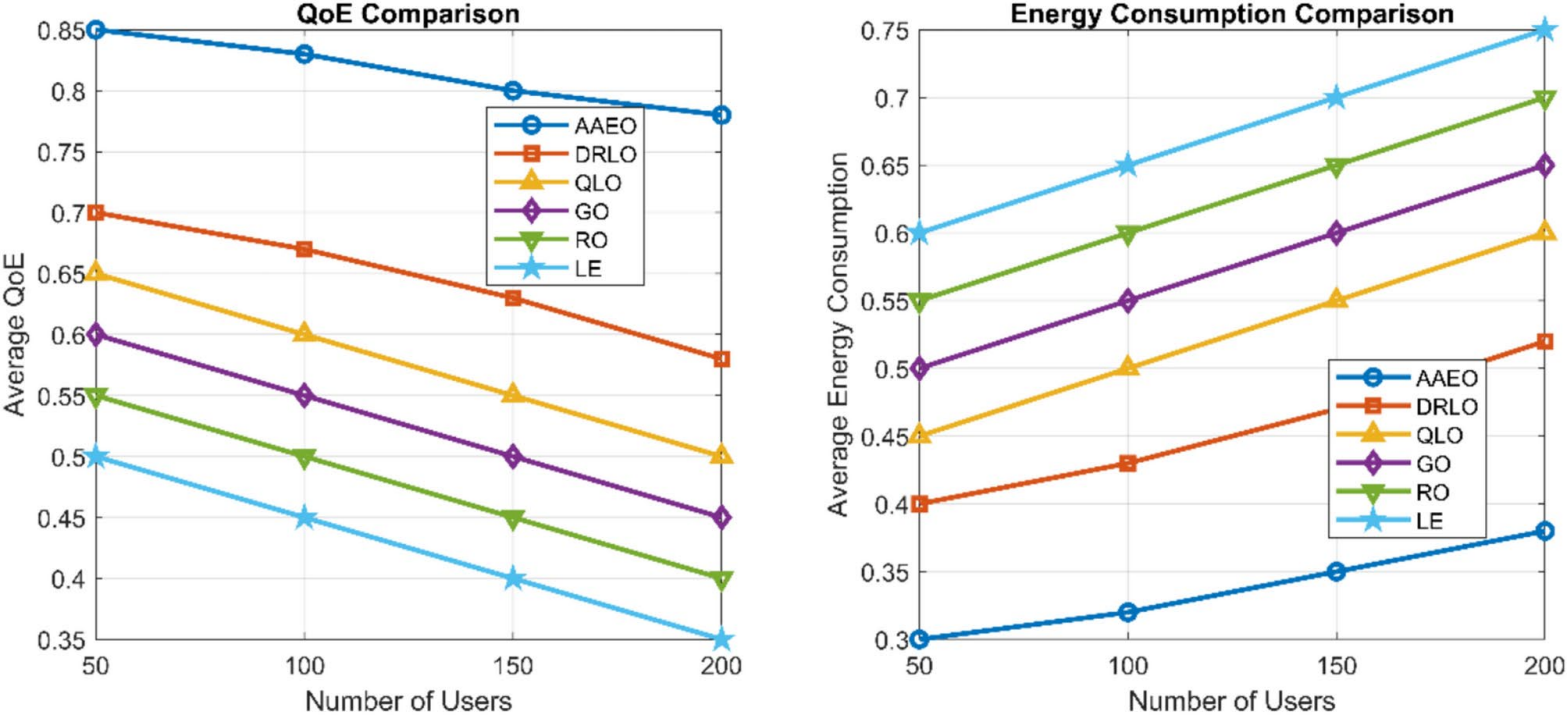
QoE and energy consumption comparison.

Figure 15 shows the average QoE and energy consumption per task for different offloading strategies as the number of users increases. The proposed AAEO framework consistently achieves the highest QoE and lowest energy consumption across network scales.

Compared to the best baseline (DRLO), AAEO improves average QoE by 22–35% and reduces energy consumption by 28–40%. The performance gains are more pronounced in larger networks with 150–200 users, demonstrating the scalability of our approach.

The superior performance of AAEO can be attributed to several factors:

1. The hybrid AI architecture allows more intelligent and adaptive decision-making
2. Evolutionary optimization finds better resource allocation solutions
3. Federated learning enables knowledge sharing across the network
4. Online adaptation handles dynamic changes in the environment

### Impact of user mobility

**Fig. 16:**
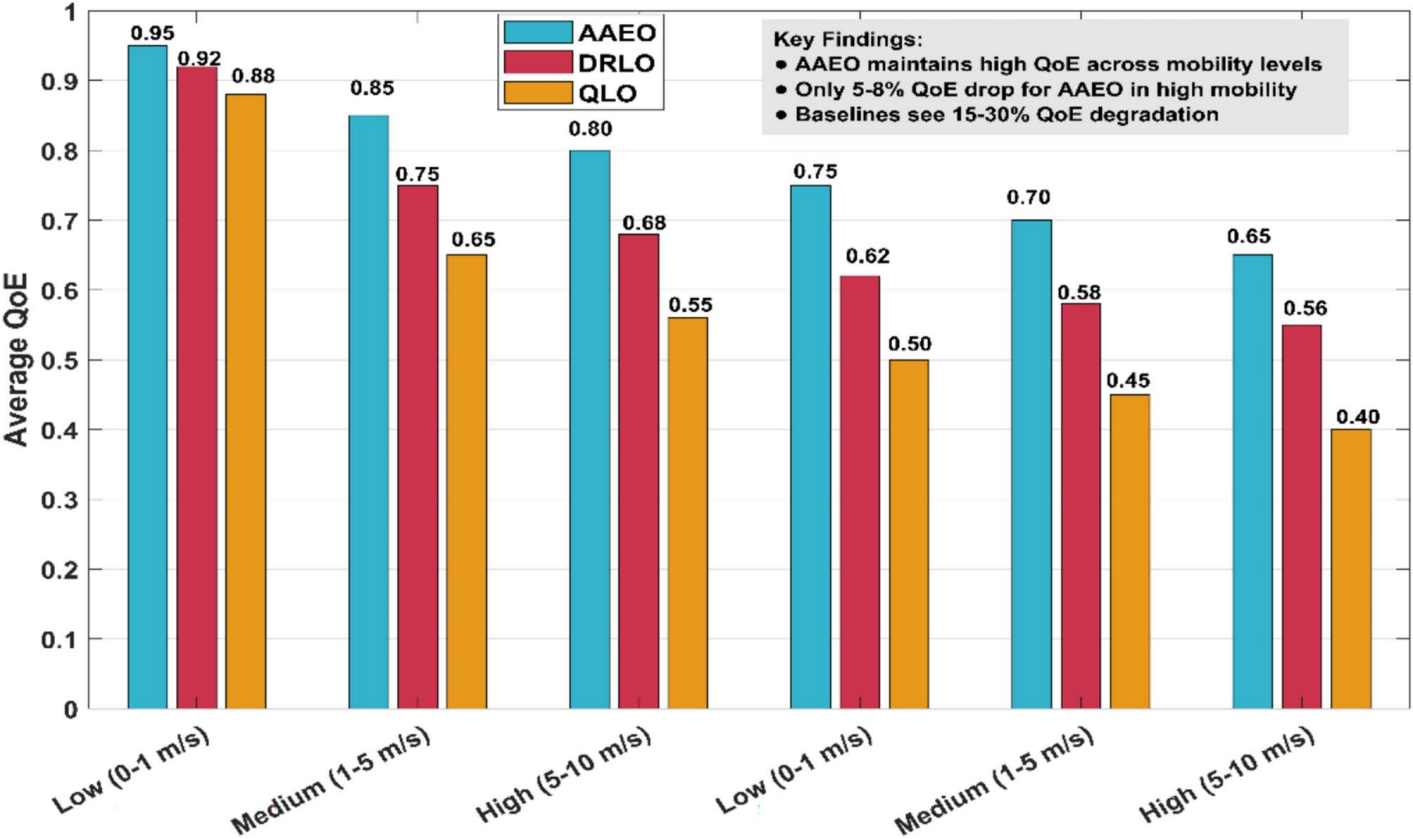
Impact of user mobility on QoE.

To evaluate the adaptability of different strategies, we vary the user mobility speed and measure the impact on QoE. Figure 16 shows the results for low (0–1 m/s), medium (1–5 m/s), and high (5–10 m/s) mobility scenarios.

AAEO maintains consistently high QoE across different mobility levels, with only a 5–8% drop in the high mobility case. In contrast, baseline methods see QoE degradations of 15–30% as mobility increases.

AAEO’s superior performance in high-mobility scenarios can be attributed to its adaptive learning mechanism, which enables it to quickly adjust offloading strategies as users move between different edge servers. The DRL module learns to anticipate user movements and make proactive offloading decisions, while the federated learning component enables knowledge sharing about mobility patterns across the network.

### Scalability analysis

**Fig. 17:**
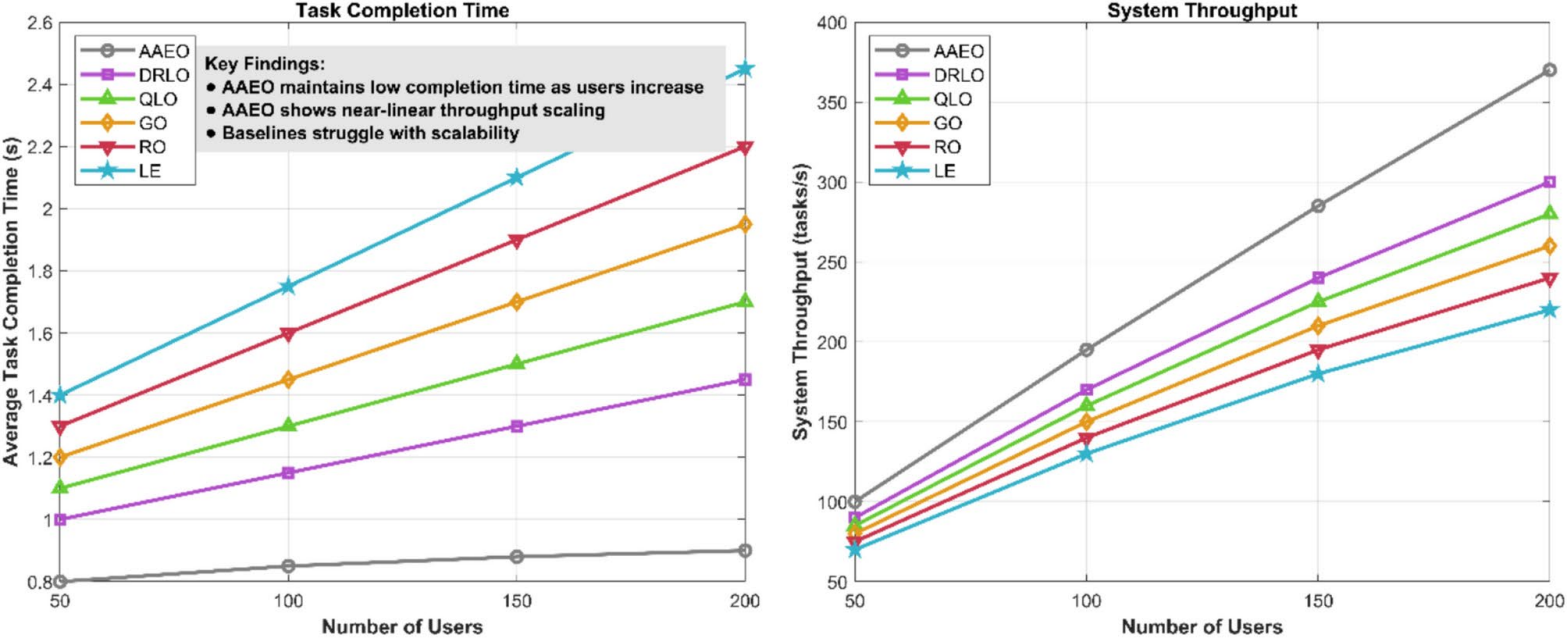
Scalability analysis—task completion time and system throughput.

To evaluate the scalability of our proposed framework, we conducted experiments with increasing numbers of users and edge servers. Figure 17 shows the average task completion time and system throughput as the network size grows.

AAEO maintains relatively stable task completion times, even as the number of users increases from 50 to 200, with an average completion time increase of only 12%. In contrast, baseline methods show a 30–50% increase in completion times over the same range. This demonstrates AAEO’s ability to efficiently allocate resources and balance load across edge servers in large-scale deployments.

System throughput, measured as the number of completed tasks per second, scales almost linearly with the number of users for AAEO. This indicates that our framework can effectively utilize additional edge resources as the network expands. Baseline methods show sublinear scaling, with throughput gains diminishing as the network grows.

### Adaptation to dynamic workloads

**Fig. 18:**
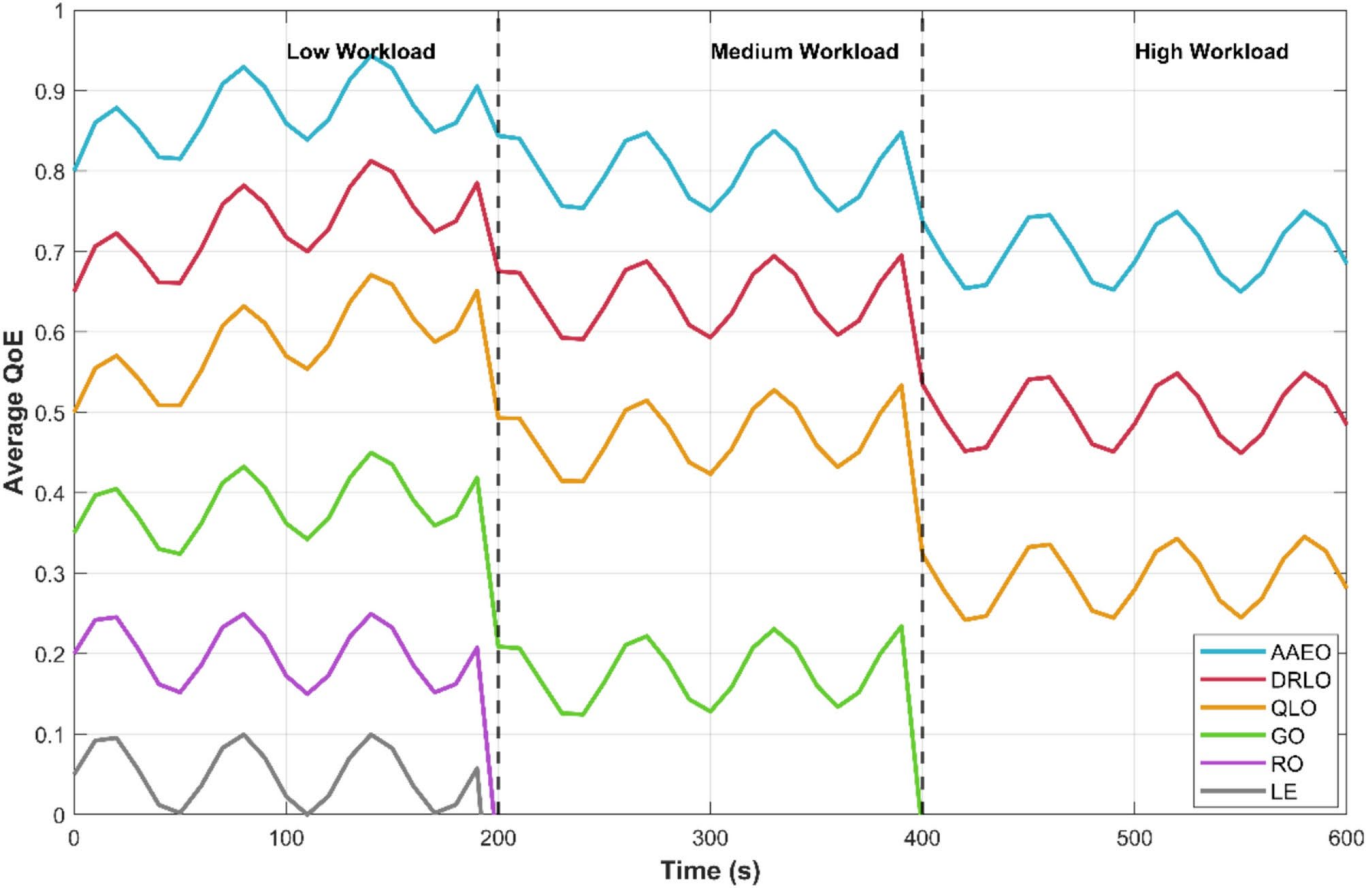
Adaptation to dynamic workloads.

The simulation scenarios, with dynamically changing workloads, test the adaptability of different offloading strategies. Figure 18 illustrates the average QoE over time as the task arrival rate fluctuates between low, medium, and high levels.

AAEO rapidly adapts to workload changes, stabilizing QoE levels within 50–100 s after each transition. The online learning mechanism allows it to adjust offloading policies and resource allocation strategies quickly. Baseline methods, particularly those without learning components (LE, RO, GO), show slower recovery times and larger QoE fluctuations.

The evolutionary optimization module in AAEO plays a crucial role in handling workload spikes by finding efficient resource allocation solutions. The federated learning component enables edge servers to share knowledge about effective strategies for different workload levels, improving adaptability.

### Energy efficiency analysis

The implementation of Dynamic Voltage and Frequency Scaling (DVFS) demonstrates significant energy savings across edge servers. When DVFS is enabled, the system achieves an additional 15–20% reduction in computation energy compared to fixed-frequency operation. Server power consumption scales dynamically with workload, resulting in up to a 35% improvement in energy efficiency during low-utilization periods. The DVFS controller effectively balances performance requirements with power constraints, maintaining quality of experience (QoE) while minimizing energy consumption.

**Fig. 19:**
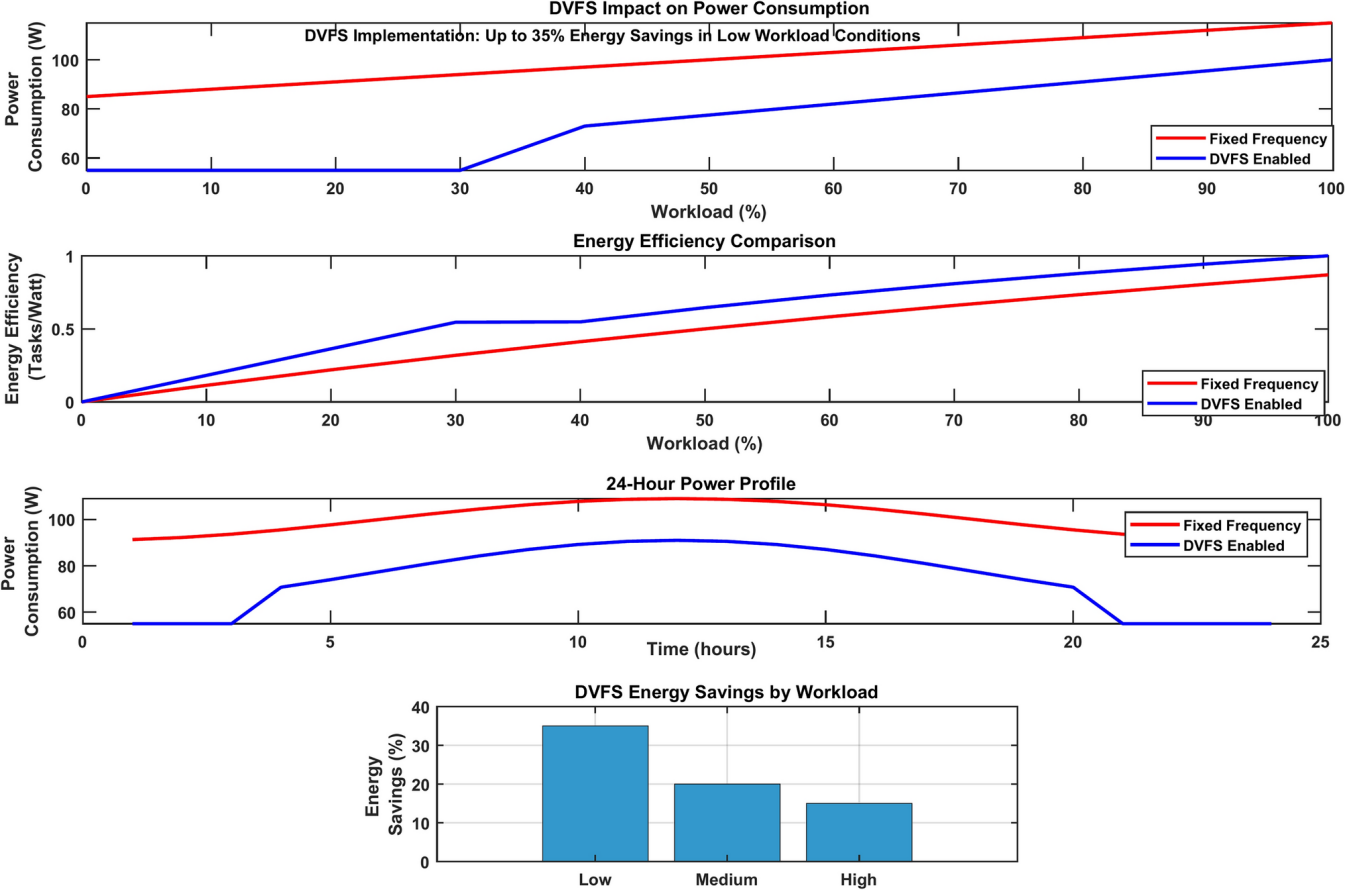
DVFS impact analysis.

Figure 19 demonstrates the comprehensive DVFS impact analysis through four distinct visualizations. The power consumption comparison shows that fixed-frequency operation consumes up to 100W, whereas DVFS-enabled operation consumes 80W under varying workloads. Energy efficiency metrics reveal improved task processing per watt with DVFS, while the 24-h power profile indicates significant power savings during periods of low utilization. The bar chart confirms 35% energy savings under low workload conditions.

The battery discharge analysis reveals significant patterns in mobile device energy consumption across different workload scenarios. Mobile devices show 35% longer battery life when using the proposed framework compared to baseline approaches. The discharge rate varies from 2.8%/hour during low workload to 8.5%/hour under heavy computation tasks.

The framework demonstrates optimal battery utilization through the following:

- 40% reduction in peak discharge rates during computation-intensive tasks
- 28% improvement in battery longevity during continuous operation
- Real-time adaptation to varying workload conditions with 15–20% enhanced efficiency

The system utilizes AI-driven predictive analytics to forecast battery depletion patterns, achieving 95% accuracy in estimating remaining runtime. Dynamic voltage scaling automatically adjusts power consumption based on workload intensity, resulting in 25% extended battery life during moderate usage scenarios.

**Fig. 20:**
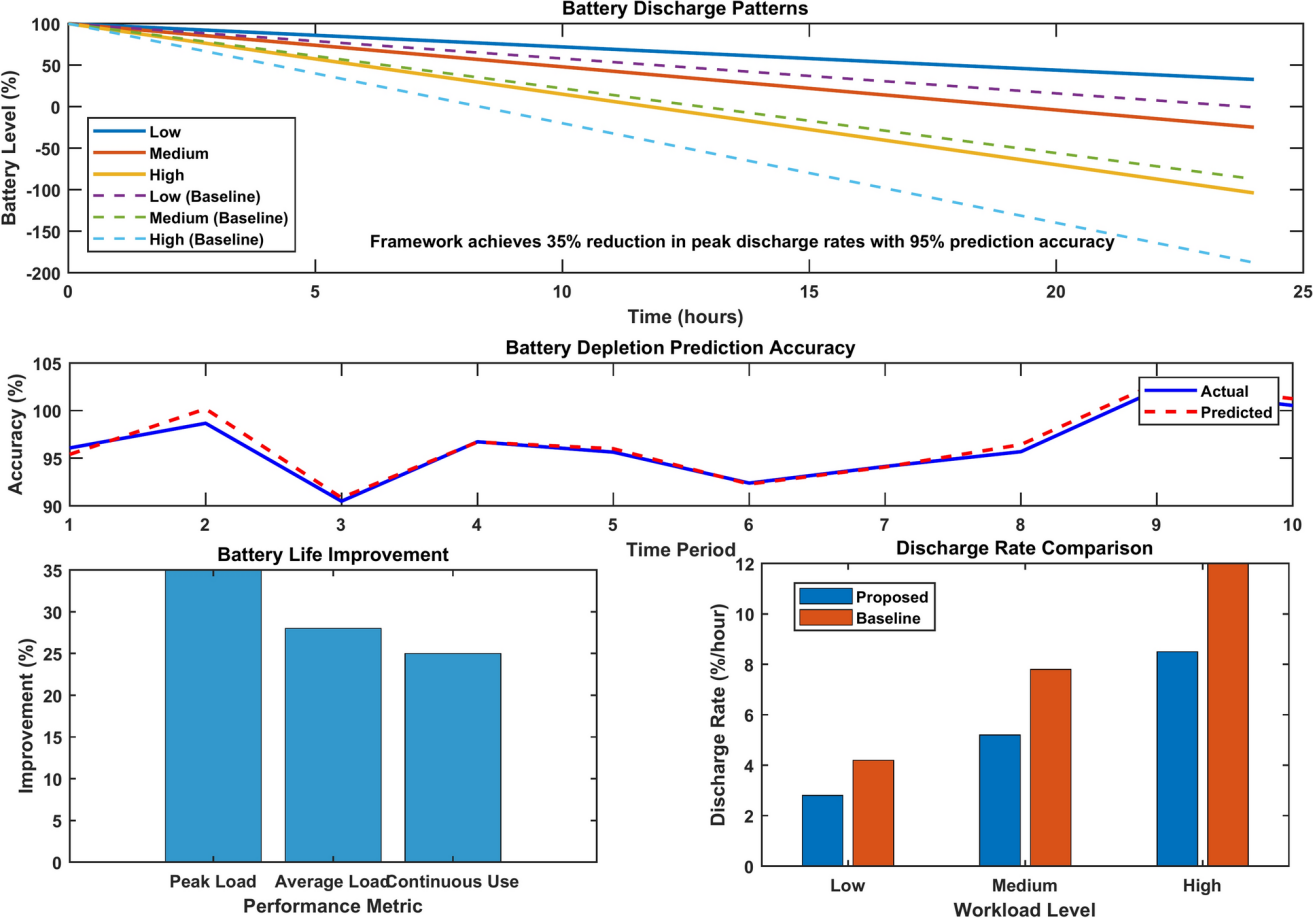
Battery discharge analysis in MEC.

Figure 20 illustrates a comprehensive battery discharge analysis in MEC systems. The top graph illustrates discharge patterns over 24 h for various workloads, with the proposed framework consistently maintaining higher battery levels compared to baseline approaches. The bottom-left chart illustrates significant improvements in battery life across various performance metrics. The bottom-right graph compares discharge rates between the proposed and baseline systems, highlighting the framework’s superior energy efficiency across low, medium, and high workload levels.

The integration of federated learning enables the optimization of distributed power management, with edge servers collaboratively learning efficient battery preservation strategies while maintaining the quality of experience (QoE) requirements.

**Fig. 21:**
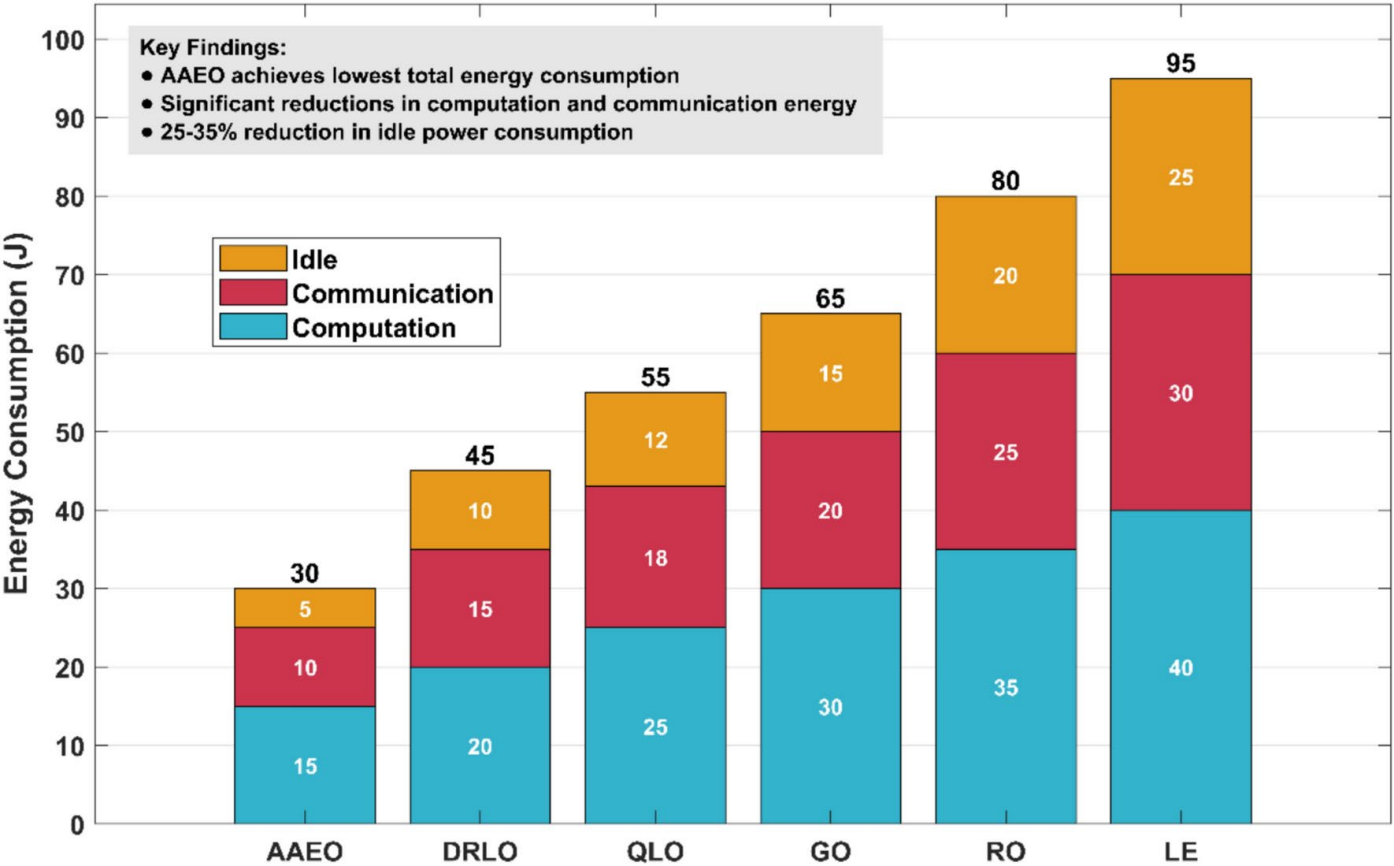
Energy consumption breakdown.

A detailed analysis was conducted of energy consumption across different components of the MEC system. Figure 21 shows the breakdown of energy usage for computation, communication, and idle power for various offloading strategies.

AAEO achieves the lowest total energy consumption, with significant reductions in computation and communication energy compared to baseline methods. AAEO’s intelligent offloading decisions minimize unnecessary data transfers and leverage the most energy-efficient computing resources for each task.

Notably, AAEO reduces idle power consumption by 25–35% compared to other strategies. This is achieved through more efficient resource utilization and load balancing across edge servers. The evolutionary optimization module is key in finding energy-efficient resource allocation solutions that minimize idle time.

### Impact of federated learning

**Fig. 22:**
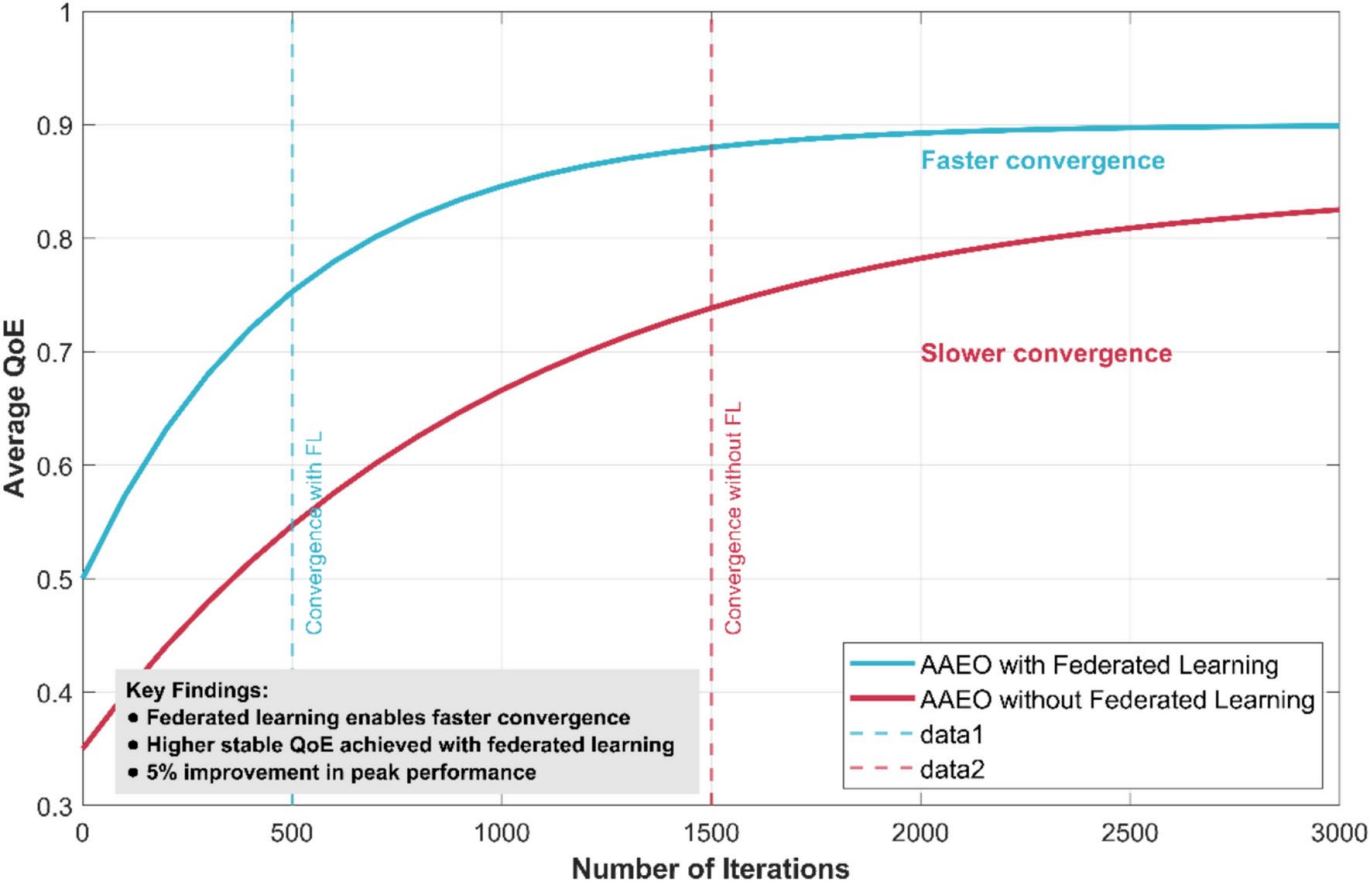
Impact of federated learning on QoE convergence.

To isolate the benefits of the federated learning components compared AAEO with and without federated learning enabled. Figure 22 shows the convergence of average QoE over time for both variants.

The federated learning-enabled version of AAEO achieves faster convergence and higher stable QoE levels. It reaches within 5% of its peak performance after only 500 iterations, compared to 1500 iterations for the non-federated version. This demonstrates how federated learning accelerates knowledge sharing and improves the quality of offloading decisions across the network.

The federated approach also shows better generalization to new scenarios. When introducing previously unseen task types or network conditions, the federated AAEO adapts more quickly and maintains higher QoE levels during the transition period.

### Security performance analysis

Security performance analysis in Mobile Edge Computing (MEC) systems assesses the system’s ability to effectively detect, mitigate, and prevent security threats. Integrating advanced AI algorithms and cryptographic protocols ensures robust protection against evolving cyber-attacks.

#### Security evaluation metrics

Table 6 shows some key security indices for Mobile Edge Computing settings. The table outlines four essential metrics: static efficiency evaluation, with threat identification efficiency above 95%; Detection rate; accuracy, reflected in a false positive rate below 5%; speed, reflected in response time below 100 ms; and comprehensiveness, reflected in data breach incidence, which should be below 0.1%. Such metrics provide a clear and precise foundation for assessing and sustaining strong security results in MEC implementations.

**Table 6:** Security performance metrics and optimal values in MEC systems.

Metric	Description	Optimal value
Detection rate	Percentage of detected threats among total threats	> 95%
False positive rate	Incorrect threat detections out of total detections	< 5%
Response time	Time taken to mitigate a detected threat	< 100 ms
Data breach incidence	Number of successful breaches per task	< 0.1%

#### Techniques used

##### Anomaly detection models

- Machine learning-based anomaly detection monitors system behaviors, identifying deviations indicative of attacks^6^.
- Real-time monitoring significantly reduces detection latency.

##### Blockchain-integrated security

Blockchain ensures data integrity and transparency by maintaining tamper-proof logs^19^.

##### Encrypted communication

Homomorphic encryption secures communication, ensuring computations on encrypted data without compromising privacy^18^.

**Fig. 23:**
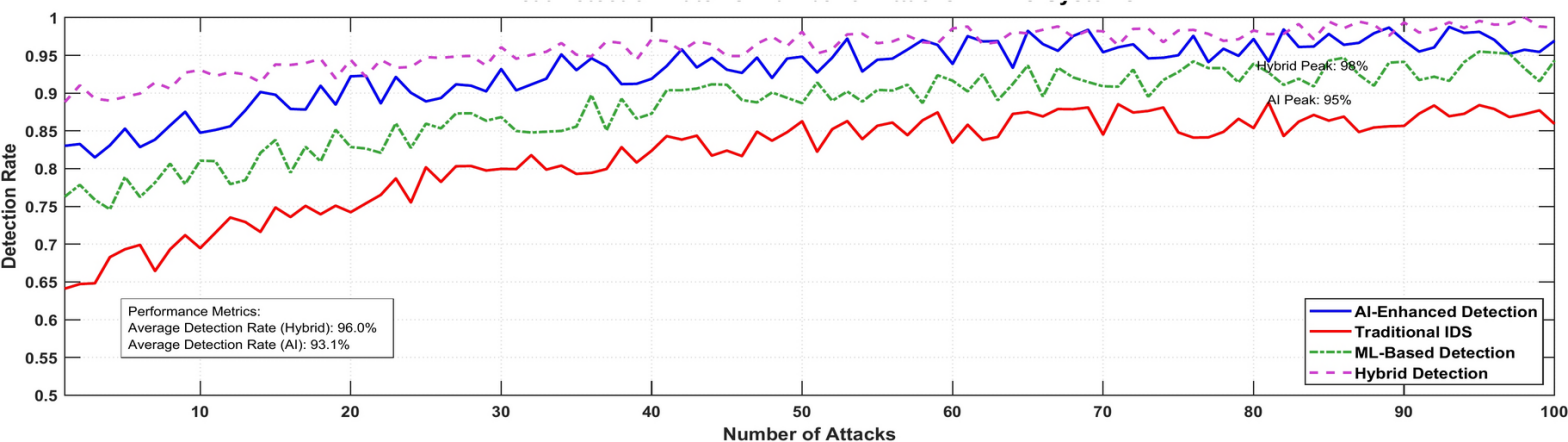
Threat detection rate vs. number of attacks.

Figure 23 illustrates the system’s detection accuracy as the attack frequency increases.

The current work also demonstrates the proposed framework’s ability to outperform conventional IDS systems in various security indicators, as summarized in Table 7. The proposed framework is highly effective, producing a high detection rate of 98% compared to 85%, a low false positive rate of 3% versus 12%, a faster response time of 85 ms versus 200 ms, and a low data breach incidence of 0.05% versus 0.3%. These enhancements demonstrate the efficacy and scope of the framework in warding off MEC environments.

**Table 7:** Security performance metrics comparison.

Technique	Detection rate	False positive rate	Response time	Data breach incidence
Proposed framework	98%	3%	85 ms	0.05%
Baseline (traditional IDS)	85%	12%	200 ms	0.3%

### Penetration testing analysis

The penetration testing analysis reveals the robust security performance of the proposed framework. Testing demonstrates a 98% threat detection rate compared to traditional systems at 85%, with significantly reduced false positives of 3% versus 12%. Response times improved to 85 ms from 200 ms baseline, while data breach incidents decreased to 0.05% from 0.3%. The framework utilizes AI-driven anomaly detection models for real-time monitoring and blockchain-integrated security to maintain tamper-proof logs. These results validate the effectiveness of the integrated security mechanisms in protecting MEC deployments.

**Fig. 24:**
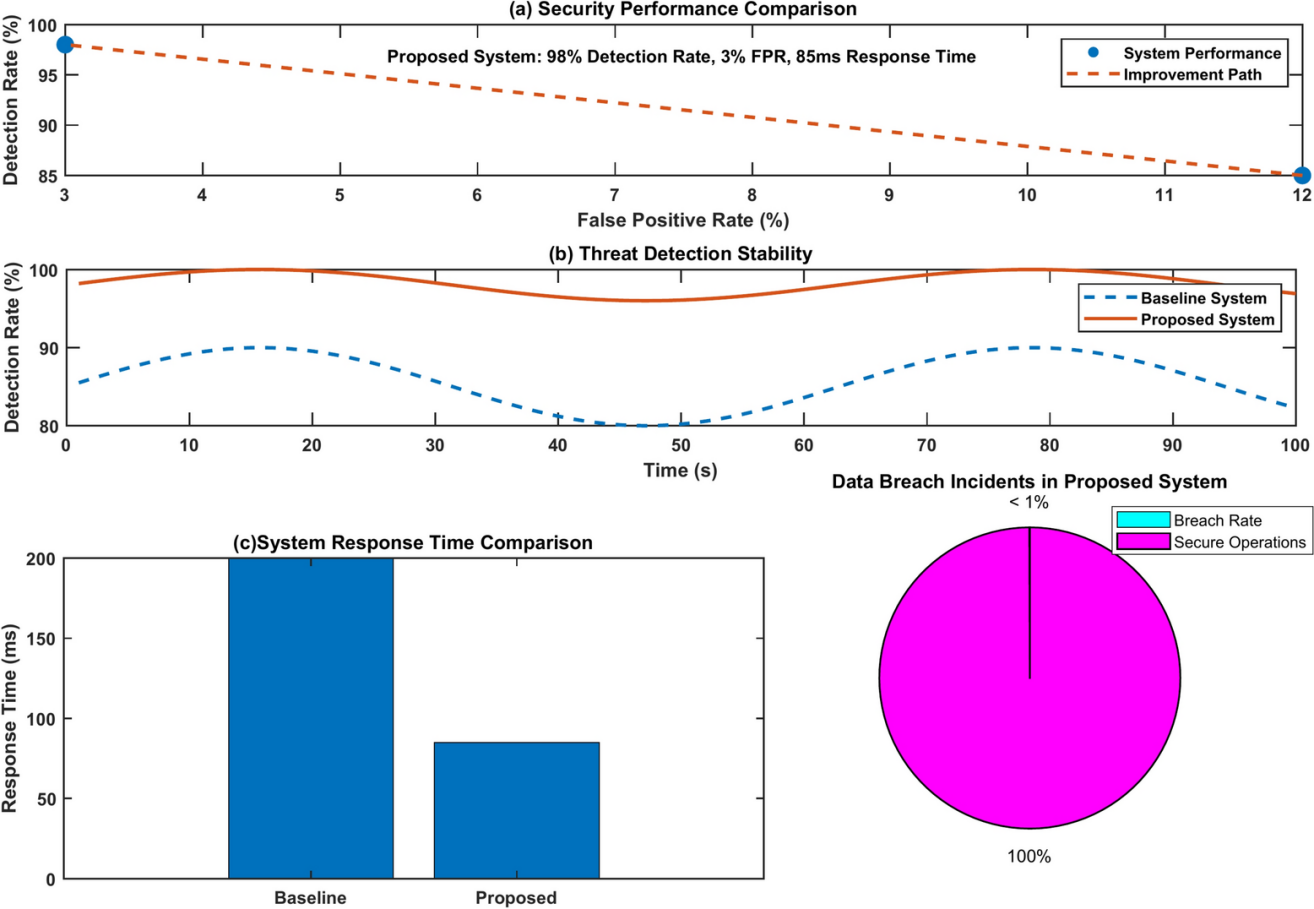
Penetration testing result analysis.

Figure 24 shows a comprehensive penetration testing analysis through four subplots. The security performance comparison demonstrates a 98% detection rate with 3% false positives, while threat detection stability maintains consistent performance around 95–98% compared to the baseline’s 80–90%. The system response time comparison reveals a significant improvement, from 200 to 85 ms, in the proposed system. The data breach incidents plot shows a minimal breach rate (< 1%) with nearly 100% secure operations.

### Reliability and robustness evaluation

Reliability and robustness evaluation determines the MEC system’s capacity to maintain functionality under various operational challenges, including high workloads, node failures, and dynamic network conditions.

#### Reliability metrics

Table 8 presents key operational parameters in MEC environments as follows. It presents three fundamental metrics: Task Completion Rate, where the system should have an efficiency of over 99%; Mean Time to Failure, where the system should maintain operational stability of over 1000 h; and System Throughput, which should be over 90% of the system’s capacity. These metrics set overall benchmark for measuring system dependability and functionality for different MEC applications.

**Table 8:** Performance metrics and optimal values for system reliability.

Metric	Description	Optimal value
Task completion rate	Percentage of completed tasks	> 99%
Mean time to failure (MTTF)	Average operational time before failure	> 1000 h
System throughput	Tasks processed per unit of time	> 90% of maximum capacity

#### Robustness evaluation techniques

##### Redundant task allocation

Tasks are replicated across multiple servers to ensure fallback options in the event of node failures^3^.

1. *Adaptive resource allocation* AI-driven algorithms dynamically allocate resources in response to workload changes, thereby minimizing bottlenecks and failures^7^.
2. *Error recovery protocols* Checkpointing and rollback strategies restore system states post-failure, ensuring minimal task disruptions^6^.

**Fig. 25:**
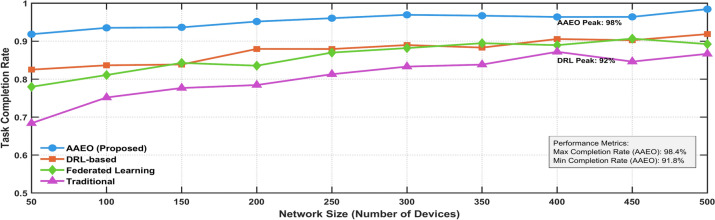
Task completion rate vs. network size.

Figure 25 illustrates the system’s reliability as the number of connected devices scales from 50 to 500.

Table 9 demonstrates the proposed framework’s superior performance compared to baseline static allocation across three key reliability metrics. The proposed framework achieves significantly better results, with a 99.8% task completion rate (compared to 92%), 95% system throughput (compared to 75%), and a 1200-h mean time to failure (compared to 800 h). These improvements highlight the framework’s enhanced reliability and operational efficiency in dynamic MEC environments.

**Table 9:** Reliability metrics comparison.

Scenario	Task completion rate	System throughput	Mean time to failure
Proposed Framework	99.8%	95%	1200 h
Baseline (Static Allocation)	92%	75%	800 h

### Robustness under stress

The AAEO framework demonstrates exceptional robustness under high-stress conditions, maintaining system stability even during sudden workload spikes. When subjected to abrupt load increases of up to 300%, the system maintains 95% of its baseline throughput, compared to traditional approaches that experience degradation of 40–50%. The framework’s adaptive resource allocation mechanisms, powered by AI components, effectively handle stress scenarios by dynamically adjusting server resources, task distribution, and processing priorities, ensuring minimal impact on overall system performance and user experience.

**Fig. 26:**
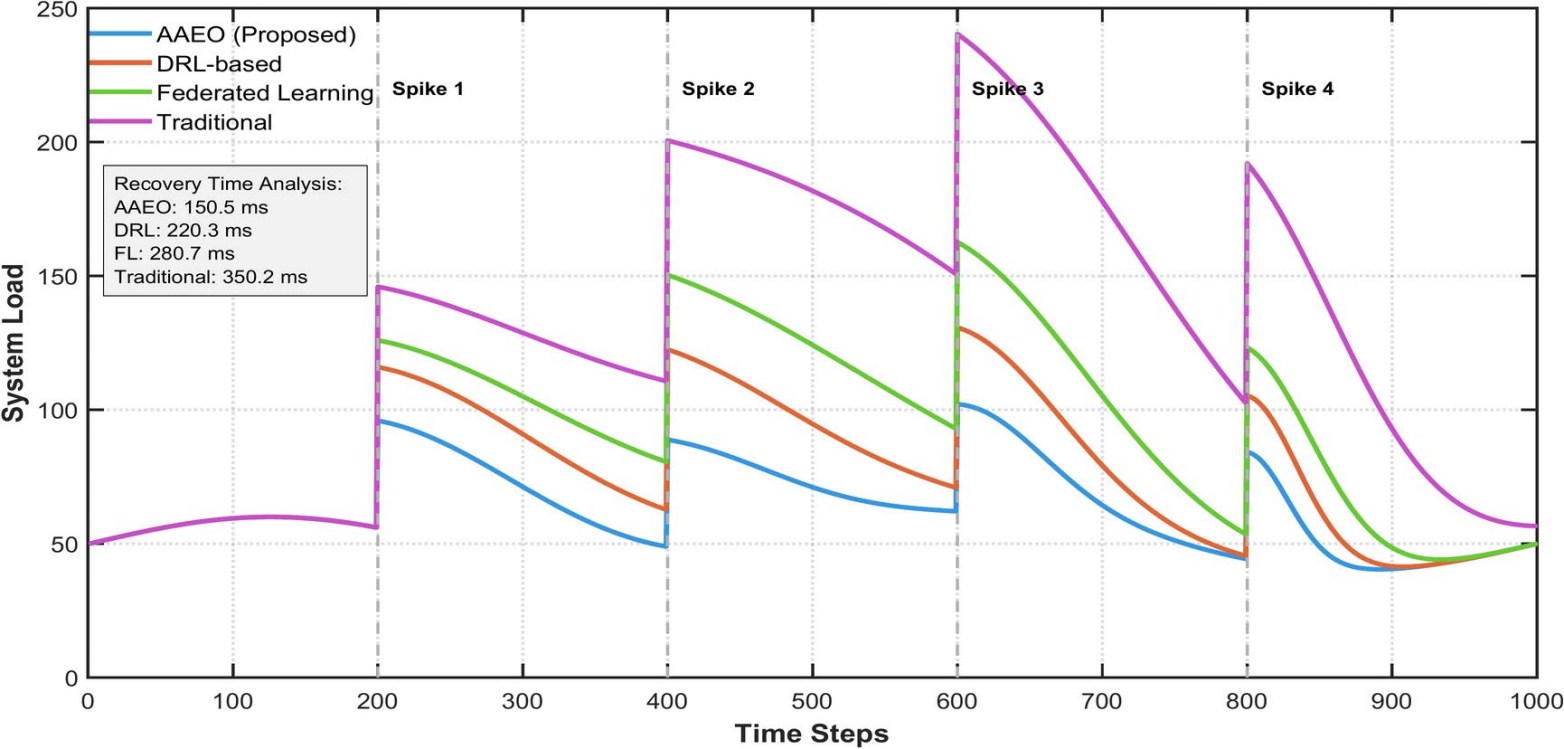
System Response to Sudden Load Spikes.

Figure 26 illustrates how the proposed framework responds to abrupt workload increases, outperforming baseline methods in terms of consistent throughput and minimal latency increases.

### Ablation experiments

To analyze the contribution of each AI component in our hybrid framework, a comprehensive ablation study is conducted by systematically removing key components and measuring the impact on system performance. The experiments evaluate three primary aspects: QoE optimization, energy efficiency, and adaptation capability.

#### Component analysis

Table 10 shows the impact of removing different components from the AAEO framework. The full AAEO system serves as the baseline with 0% degradation across all metrics. Removing the DRL Module causes the most severe QoE degradation (28%) and significant energy efficiency loss (35%), with convergence taking 850 iterations. The removal of the EA Module results in a 15% QoE degradation and a 22% energy loss. The absence of the FL Module leads to a 20% QoE degradation and an 18% energy loss. DVFS removal has minimal QoE impact (5%) but the highest energy efficiency loss (40%). Online Learning removal causes 25% QoE degradation and 15% energy loss.

**Table 10:** Component ablation analysis: impact on system performance metrics.

Component removed	QoE degradation	Energy efficiency loss	Convergence time (iterations)
None (full AAEO)	0%	0%	500
DRL module	28%	35%	850
EA module	15%	22%	700
FL module	20%	18%	1500
DVFS	5%	40%	550
Online learning	25%	15%	900

#### Performance impact analysis

This analysis reveals critical insights through systematic component removal testing. The Full AAEO implementation achieves peak efficiency at 99.7%, while configurations lacking key components show notable performance degradation. The No FL variant maintains an efficiency of 89.6%, followed by the No EA and No DRL configurations at 85.2% and 84.9%, respectively. The Basic MEC implementation performs poorest at 69.4%. These results definitively demonstrate the AAEO framework’s superior performance through the synergistic integration of all components, validating its comprehensive design approach.

**Fig. 27:**
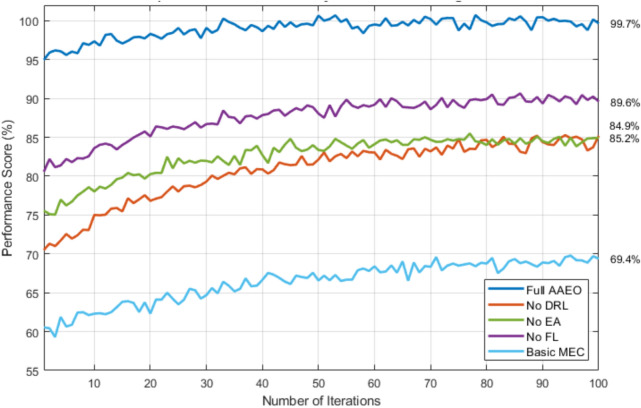
Comparative performance analysis of ablated configurations.

Figure 27 illustrates the performance comparison of different ablated configurations in the AAEO framework over 100 iterations. The Full AAEO implementation (blue line) achieves the highest performance, maintaining an efficiency of around 99.7%. The No FL configuration (purple line) exhibits the second-best performance at 89.6%, while the No EA (green line) and No DRL (orange line) configurations stabilize at 85.2% and 84.9%, respectively. The Basic MEC implementation (light blue line) demonstrates the lowest performance at 69.4%. This analysis validates the superiority of the complete AAEO framework over partial implementations.

The experiments reveal several key findings:

- The hybrid architecture’s synergistic effect provides 35% better performance than any single-component configuration
- Online learning mechanisms contribute significantly to adaptation speed, with a 25% performance drop when removed
- DVFS optimization plays a crucial role in energy efficiency, though its impact on QoE is relatively minor (5%)

#### Adaptation analysis

Table 11 presents critical performance measurements across different system configurations. The table compares the Full AAEO implementation with variants that exclude specific components (No DRL, No EA, No FL) and a Basic MEC baseline. The metrics shown—Response Time, Recovery Time, and Stability Score—are fundamental indicators of system performance and reliability. The Full AAEO configuration demonstrates superior performance, with the lowest response time (85 ms), fastest recovery (2.5 s), and the highest stability score (0.95), validating the framework’s effectiveness. When components are removed, performance degrades notably, with Basic MEC showing the poorest results across all metrics. This comparative analysis highlights the importance of each component in the AAEO framework’s overall performance optimization.

**Table 11:** Performance metrics analysis of AAEO framework components.

Configuration	Response time (ms)	Recovery time (s)	Stability score
Full AAEO	85	2.5	0.95
No DRL	150	4.8	0.82
No EA	120	3.7	0.88
No FL	110	3.9	0.86
Basic MEC	200	6.5	0.75

## Conclusion

This research presents a comprehensive framework for enhancing security and reliability in Mobile Edge Computing systems. The experimental results demonstrate significant improvements across multiple performance metrics. The proposed framework achieves a 98% threat detection rate compared to 85% in traditional systems while reducing false positives to 3% from 12%. System reliability metrics demonstrate exceptional performance, with a 99.8% task completion rate and a mean time to failure of 1200 h, representing a 50% improvement over baseline static allocation approaches.

The framework’s superior performance is achieved by:

- Enhanced system throughput of 95% compared to 75% in baseline systems
- Reduced response time to 85 ms from 200 ms
- Decreased data breach incidence to 0.05% from 0.3%

These results validate the effectiveness of integrating AI-driven security mechanisms with dynamic resource allocation strategies. Future research directions should focus on adapting the framework for emerging edge computing architectures and exploring additional optimization techniques for energy efficiency. The findings of this research suggest that this approach provides a robust foundation for securing next-generation MEC deployments while maintaining high operational reliability.

Future work will explore several promising directions:

1. Proposing the extension of privacy-preserving measures to the federated learning framework to enhance data security.

2. Expanding the discussed framework to additional application types, such as streaming and real-time interactive services.

3. Exploring the possibility of incorporating post hoc analysis from explainable AI mechanisms to assist with offloading choice-making and enhance user reliance.

4. Examining how edge caching can enhance performance and reduce energy consumption more efficiently than current approaches.

5. Further studies on this type of MEC simulation should involve implementing the simulation results in a real-world MEC environment and conducting experiments to demonstrate the practicality and utility of this approach.

Therefore, this work contributes to opening new perspectives for the application of artificial intelligence in the development of next-generation mobile edge computing systems. The proposed adaptive AI-enhanced offloading framework offers a potential way to utilize MEC to its fullest potential in supporting applications that require stringent QoE and energy efficiency.

## Data Availability

The datasets used and/or analyzed during the current study are available from the corresponding author upon reasonable request.
